# Microtubule-associated ROP interactors affect microtubule dynamics and modulate cell wall patterning and root hair growth

**DOI:** 10.1242/dev.200811

**Published:** 2022-11-16

**Authors:** Gil Feiguelman, Xiankui Cui, Hasana Sternberg, Eliran Ben Hur, Takeshi Higa, Yoshihisa Oda, Ying Fu, Shaul Yalovsky

**Affiliations:** ^1^ School of Plant Sciences and Food Security, Tel Aviv University, Tel Aviv 6997801, Israel; ^2^ State Key Laboratory of Plant Physiology and Biochemistry, College of Biological Sciences, China Agricultural University, Beijing 100193, China; ^3^ Department of Gene Phenomics and Function, National Institute of Genetics, Mishima, Shizuoka 411-8540, Japan; ^4^ Department of Genetics, School of Life Science, The Graduate University for Advanced Studies, SOKENDAI, Mishima, Shizuoka 411-8540, Japan; ^5^ Joint Laboratory for International Cooperation in Crop Molecular Breeding, Ministry of Education, Beijing 100193, China

**Keywords:** ROP, Microtubules, Cell wall, Root hair, Metaxylem, Protoxylem

## Abstract

Rho of plant (ROP) proteins and the interactor of constitutively active ROP (ICR) family member ICR5/MIDD1 have been implicated to function as signaling modules that regulate metaxylem secondary cell wall patterning. Yet, loss-of-function mutants of ICR5 and its closest homologs have not been studied and, hence, the functions of these ICR family members are not fully established. Here, we studied the functions of ICR2 and its homolog ICR5. We show that ICR2 is a microtubule-associated protein that affects microtubule dynamics. Secondary cell wall pits in the metaxylem of *Arabidopsis icr2* and *icr5* single mutants and *icr2 icr5* double mutants are smaller than those in wild-type Col-0 seedlings; however, they are remarkably denser, implying a complex function of ICRs in secondary cell wall patterning. ICR5 has a unique function in protoxylem secondary cell wall patterning, whereas *icr2*, but not *icr5*, mutants develop split root hairs, demonstrating functional diversification. Taken together, our results show that ICR2 and ICR5 have unique and cooperative functions as microtubule-associated proteins and as ROP effectors.

## Teaser

**Summary:** The ROP interactors ICR2 and ICR5 are microtubule-associated proteins that have unique and redundant functions in secondary wall patterning in the xylem and root hair polar growth.

## INTRODUCTION

Plant microtubules function dynamically to regulate cellular functions related to cell division, cell growth, cell shape formation, pathogen invasion and abiotic stresses. Microtubule dynamics, including extension, shrinkage, catastrophe and rescue, have been studied in plant cells (Ehrhardt and Shaw, 2006; Elliott and Shaw, 2018; Shaw et al., 2003). Although plant cells have unique microtubule structures and organization, several mechanisms underlying microtubule dynamics are conserved in eukaryotes (Hamada, 2014a). Microtubules organize in several typical structures in the course of the cell cycle. During interphase, microtubules form cortical arrays beneath the plasma membrane. In plant cells, in contrast to animal and yeast cells, interphase microtubules organize without a microtubule-organizing center and their plus and minus ends are distributed throughout the cell cortex (Ehrhardt, 2008; Ehrhardt and Shaw, 2006; Elliott and Shaw, 2018; Wasteneys and Ambrose, 2009; Yagi et al., 2018; Yi and Goshima, 2018).

The dynamic nature of cortical microtubules and their ability to respond to diverse stimuli is governed by microtubule-associated proteins (MAPs), which regulate their nucleation, stability, crosslinking, severing, membrane interaction and orientation (Hamada, 2014b). For example, movement of cellulose synthases (CesAs) in the membrane is driven by the synthesis of cellulose chains and overlays with cortical microtubules (Paredez et al., 2006). Cortical microtubules have been suggested to affect CesA localization in the plasma membrane and to regulate their movement (Gutierrez et al., 2009). Several MAPs are known to mediate CesA and microtubule colocalization (Bringmann et al., 2012; Endler et al., 2015; Gu et al., 2010; Kesten et al., 2019; Lei et al., 2013; Li et al., 2012).

Microtubule organization and dynamics are coordinated by a variety of MAPs in time and space. This spatial and temporal regulation is dependent on the function of Rho family of small G proteins called Rho of Plants (ROPs). ROPs are the plant-specific subfamily of the Rho family of small G proteins. ROPs function as plasma membrane-anchored molecular switches that cycle between active, GTP-bound, and inactive, GDP-bound states (Feiguelman et al., 2018). In the active, GTP-bound state, ROPs interact with target effector proteins to perform their biological functions (Dvorsky and Ahmadian, 2004; Feiguelman et al., 2018). ROPs regulate a variety of cellular processes such as the organization and dynamics of the actin and microtubule cytoskeleton, endocytosis and exocytosis, and the activation of NADPH oxidase and intracellular kinase cascades. ROPs regulate cell growth and shape, cytokinesis, subcellular protein localization and responses to pathogens and abiotic stresses (Bloch and Yalovsky, 2013; Feiguelman et al., 2018; Kawano et al., 2014; Nagawa et al., 2010; Oda and Fukuda, 2014; Rivero et al., 2017; Yalovsky et al., 2008; Yang, 2008).

We previously identified a family of microtubule-associated ROP interactors that we designated ‘interactors of constitutively active ROP’ (ICRs) (Lavy et al., 2007). ICRs are coiled coil domain-containing proteins that do not contain additional known structural or catalytic domains (Lavy et al., 2007). They contain two conserved sequence motifs: an N-terminal QEEL and a C-terminal QWRKAA (Lavy et al., 2007). ICRs are subdivided into two clades, which differ in molecular mass. In *Arabidopsis*, ICR1 (AT1G17140, 38 kDa) and ICR4 (AT1G78430, 36 kDa) represent the lower molecular mass clade, whereas ICR2 (AT2G37080, 65 kDa), ICR3 (AT5G60210, 63 kDa) and ICR5 (AT3G53350, 45 kDa) represent the higher molecular mass clade.

ICR1 was characterized as a MAP that integrates ROP and Ca^2+^ signaling. It functions as a ROP-associated scaffold that interacts with a specific group of proteins (Hazak et al., 2010, 2019, 2014; Lavy et al., 2007). ICR1 is recruited to the plasma membrane by ROPs and subsequently recruits the EF-hand calcium-binding protein CALCIUM-DEPENDENT MODULATOR OF ICR1 (CMI1) to cortical microtubules. This affects CMI1 subcellular distribution and influences its function (Hazak et al., 2019; Lavy et al., 2007).

ICR5 [also known as ROP-INTERACTING PARTNER 3 (RIP3) and MICROTUBULE DEPLETION DOMAIN 1 (MIDD1)] was shown to be a MAP that interacts with the microtubule-destabilizing kinesin KINESIN-13A (Mucha et al., 2010). Functional analysis of dedifferentiating tracheary elements showed that ICR5 regulates secondary cell wall deposition in differentiating metaxylem cells through an association with depolymerizing cortical microtubules in future secondary cell wall pits (Oda and Fukuda, 2012, 2013; Oda et al., 2010). It was proposed that ICR5 is recruited to plasma membrane domains by ROP11, where it promotes local microtubule breakdown, which in turn results in the formation of cell wall pits (Oda and Fukuda, 2012, 2013). A recent study showed that ICR2 and ICR5 interact with the AGC1.5 protein kinase, which in turn phosphorylates ROPGEF4 and ROPGEF10 to promote root hair growth (Li et al., 2020).

ICR proteins are MAPs (Hazak et al., 2019; Mucha et al., 2010; Oda and Fukuda, 2012, 2013; Oda et al., 2010), yet their effect on microtubule organization is not well understood. In this work, we characterized the functions of ICR2 and ICR5 and analyzed the *icr2 icr5* single- and double-mutant phenotypes. Our results indicate that ICR2 function is associated with microtubule organization and dynamics. Additionally, the function of ICR5 in differentiating metaxylem cells is more complex than what was previously proposed.

## RESULTS

### ICR2 expression pattern

To analyze the expression pattern of the ICR2 protein, the sequence of the *ICR2* gene, including the 2225 bp of the upstream promoter sequence, was fused to the sequence encoding the β-glucuronidase (GUS) reporter (*pICR2::ICR2_genomic_*-*GUS*). At 7 days after germination (DAG), ICR2 expression was observed near the root tip, specifically in the cell division zone, and in lateral root initials, lateral roots, vascular tissues and root hairs (Fig. S1A-D). In the hypocotyl and the cotyledons, ICR2-GUS expression was strong in vascular tissues, leaf primordia and stomata linage cells (Fig. S1E-F). Although detectable, the expression was lower in mature guard cells than in stomata linage cells (Fig. S1G,H). In reproductive organs, ICR2-GUS was observed in developing floral tissue, the vasculature of pedicels and receptacles, sepals, the stamen filament, ovary and ovules, and developing seeds and siliques (Fig. S1I-L).

In agreement with the ICR2-GUS reporter data, gene expression data from the *Arabidopsis* eFP Browser (https://bar.utoronto.ca/efp/cgi-bin/efpWeb.cgi; Winter et al., 2007) indicate that *ICR2* expression is higher in the shoot apex and in seeds than in other tissues and higher during flower development than in other stages (Fig. S2). A co-expression analysis using GENEVESTIGATOR (https://genevestigator.com/; Hruz et al., 2008) indicated that the expression of *ICR2* is highly correlated with various MAPs and actin-associated proteins (Table S1). The co-expression data suggested that *ICR2* is involved in cytoskeletal organization throughout all stages of the cell cycle. Although many of the co-expressed genes are uncharacterized, the strong correlations of *ICR2* levels with levels of *ICR3* and *ICR4* suggest that they either function together or that there is some functional redundancy among these ICR family members. Interestingly, *MAP65-2*, the expression of which is most highly correlated with that of *ICR2*, encodes a coiled coil-containing microtubule-stabilizing protein involved in microtubule bundling of both interphase and cytokinetic microtubule arrays (Guo et al., 2009; Lucas et al., 2011; Lucas and Shaw, 2012). Recently published single-cell RNA sequencing data further show that *ICR2* is expressed in the xylem, initial cells and dividing cells (https://bioit3.irc.ugent.be/plant-sc-atlas/; Graeff et al., 2021). The GUS reporter expression data are in line with the transcriptomic data and indicate that *ICR2* is highly expressed in the meristem and dividing cells, developing stomata, flower organs, ovules and seeds. The relatively high expression detected in vascular tissues and root hairs suggests that ICR2 may function in these tissues and cells.

### Generation of single and double mutants of *ICR2* and *ICR5*

In order to characterize the function of ICR2, mutants were either obtained or generated. The *icr2-1* (*GK567F02*), *icr2-2* (*GK281B01*) and *icr2-3* (*GK159B08*) T-DNA mutants, which are part of the GABI-Kat seed stock (Kleinboelting et al., 2012), were obtained from the European *Arabidopsis* Stock Centre (NASC) and are from the Columbia-0 (Col-0) background (Fig. S3). Furthermore, multiplex genome editing by CRISPR/Cas9 (Bortesi and Fischer, 2015) was carried out in order to generate multiple mutant alleles in *ICR2* and *ICR5*. We identified two independent *icr5* single-mutant alleles and two independent *icr2 icr5* double-mutant alleles (Fig. S4). The CRISPR/Cas9-mediated genome editing generated indels resulting in mutant genes encoding truncated proteins in which most residues are missing (23 missing residues out of 584 in the two *icr2* CRISPR alleles, and 11, 23 and 31 missing residues out of 397 in the *icr5* CRISPR alleles). The predicted truncated proteins lack the coiled coil domains and part of the N-terminal microtubule-binding domain, and are, therefore, very likely inactive. Hence, all mutants were considered nulls. Reverse transcription PCR (RT-PCR) showed that expression of *ICR5* mRNA was reduced to negligible levels in the *icr5-1* and *icr5-2* mutants and to lower levels in the *icr5-3* mutant (Fig. S4D).

### ICR2 and ICR5 contribute to metaxylem pit formation

**Fig. 1.**
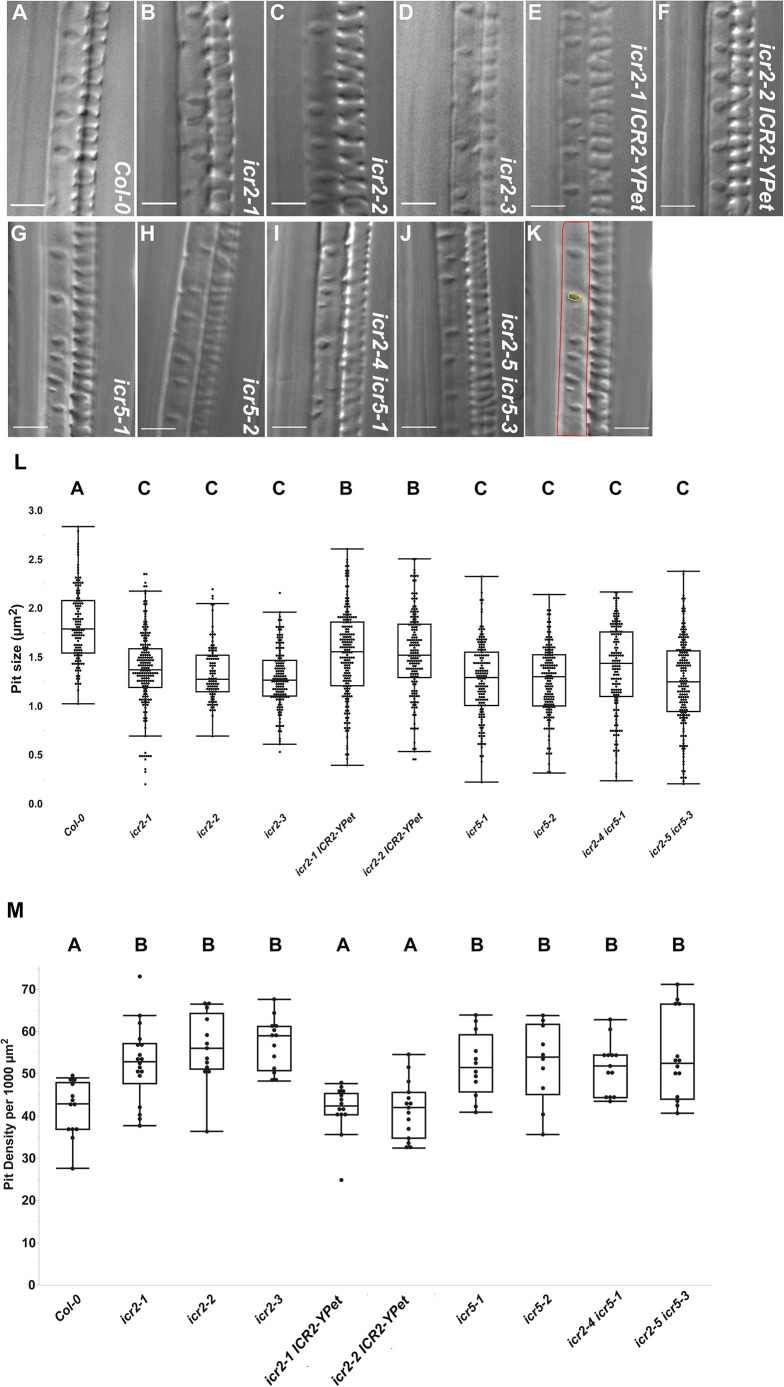
**ICR2 and ICR5 function in metaxylem secondary cell wall pit size and density.** (A-K) Representative DIC images of seedlings at 8 DAG, taken three or four cells shootward from the initiation of metaxylem differentiation. Scale bars: 5 μm. (K) Examples of pit area (yellow) and density (number of pits divided by the area of red polygon) measurements using the image from panel G. Scale bar: 5 μm. (L) Mean pit area (µm^2^). *n*>101 pits. (M) Pit density (number of pits per 1000 µm^2^). *n*>10 metaxylem cells. Statistical analyses were performed by one-way ANOVA with Tukey's HSD post hoc test. For panel L, *F* (10, 1716)=38.19, *P*<0.0001; and for panel M, *F* (10, 137)=9.2886, *P*<0.0001. The means with different letters are significantly different (Tukey's HSD, *P*<0.05). The boxes are the interquartile ranges, the whiskers represent the first and fourth quartiles, and the lines are the averages. Experiments were repeated twice. See Table S9 for the data shown in Fig. 1L,M.

Previous work indicated that ICR5 is required for the formation of secondary cell wall pits in the metaxylem (MX) (Oda and Fukuda, 2012; Oda et al., 2010), but the phenotype of an *icr5* mutant has not been previously described. The creation of *icr2* and *icr5* single- and double-mutant plants enabled analysis of the functions of these ICRs in MX pit formation (Fig. 1).

The analysis of *icr2* and *icr5* single mutants revealed that they have significantly smaller and denser pits than Col-0 plants (Fig. 1A-D,G,H,K). The reduction in pit size in the *icr5* background (Fig. 1L) agrees with the proposed function of ICR5 in pit formation (Oda and Fukuda, 2012; Oda et al., 2010). The results further indicate that ICR2 has a similar role to that of ICR5 in pit formation. The increase in pit density in both *icr2* and *icr5* mutant backgrounds (Fig. 1M) suggests that ICR2 and ICR5 function might be more complex and possibly also involve restriction of active ROP domains. Interestingly, the pit densities of the *icr2 icr5* double mutants (Fig. 1I,J) were not significantly different compared with those of *icr2* and *icr5* single mutants (Fig. 1M). Importantly, pit size and density were partially complemented in double transgenic plants expressing a genomic clone of *ICR2* (Fig. 1E,F,L,M). ICR2 was fused to three repeats of the YFP variant YPet, under regulation of the *ICR2* promoter. The plants also expressed the microtubule marker RFP-MBD (*icr2-1 UBQ10::RFP-MBD ICR2-3×YPet* and *icr2-2 UBQ10::RFP-MBD ICR2-3×YPet*). Collectively, the data show that ICR2 and ICR5 contribute to pit size and affect pit density (Fig. 1L,M).

### ICR5, but not ICR2, is involved in protoxylem secondary cell wall deposition

**Fig. 2.**
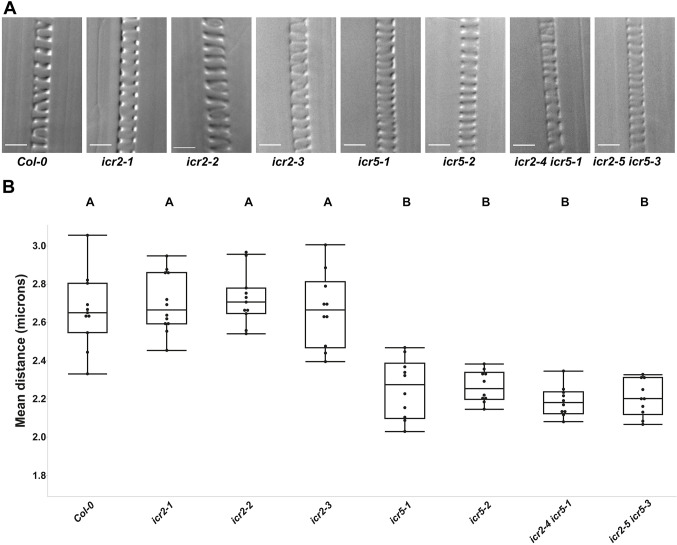
**ICR5 functions in protoxylem secondary cell wall coil patterning.** (A) Representative DIC images of protoxylem cells, imaged three or four cells shootward for initiation of differentiation. Scale bars: 5 μm. (B) Mean distance in micrometers between the lignin coils. Statistical analysis was performed by one-way ANOVA with Tukey's post hoc test. *F* (8, 86)=34.4972, *P*<0.0001. Means with different letters are significantly different (Tukey's HSD, *P*<0.05). The boxes are the interquartile ranges, the whiskers represent the first and fourth quartiles, and the lines are the averages. *n*=10 protoxylem cells for each line. Experiments were repeated twice. See Table S10 for the data shown in Fig. 2B.

To analyze whether ICR2 and ICR5 have additional roles during vascular differentiation, specifically in secondary cell wall deposition, the density of developing protoxylem (PX) lignin coils was measured (Fig. 2A). The PX lignin coils in *icr2* mutants were similar to those of Col-0, whereas the *icr5* single mutants as well as the *icr2 icr5* mutants had denser lignin deposition than that in Col-0 (Fig. 2B). This finding indicated that ICR5 is involved in secondary cell wall deposition in the PX. As there was no additive phenotype in double mutants, we reason that ICR2 does not function in the PX secondary cell wall patterning. Similar to pit formation in the MX, secondary cell wall coils still formed in the *icr5* PX, indicating that ICR5 contributes to secondary cell wall patterning in the PX, but there are additional ICR5-independent mechanisms.

### ICR2, ICR3 and ICR5 do not form homodimers or heterodimers

Coiled coil domain-containing proteins often dimerize, and it was previously shown by yeast two-hybrid and bimolecular fluorescence complementation (BiFC) assays in plants that ICR1 homodimerizes but does not form heterodimers with ICR2 (Lavy et al., 2007). Hence, we examined whether ICR2, ICR3 and ICR5, which belong to the higher molecular mass ICR clade, homodimerize or heterodimerize. BiFC assays were carried out to examine homodimerization and heterodimerization of ICR2, ICR3 and ICR5. The assays included all possible combination of the proteins fused to either the N-terminal half of YFP (YN) or the C-terminal half of YFP (YC). In all the assays, only autofluorescence was detected upon λ scan fluorescence acquisition between 521 and 690 nm. When the fluorescence was unmixed for dye separation of YFP fluorescence, no signal was detected (Fig. S5). Importantly, YFP signals were observed when the interaction of ICR2, ICR3 or ICR5-YC with several ROPs was tested (Fig. 4; Fig. S8). Homodimerization and heterodimerization of ICR2 and ICR5 with each other and with ICR3 were also examined by yeast two-hybrid assays. In agreement with the results of the BiFC assays, no homodimerization or heterodimerization were detected (Fig. S6). Interestingly, the data also show that unlike ICR1 (Lavy et al., 2007), ICR2, ICR3 and ICR5 do not form homodimers.

### *icr2* mutants develop split and altered root hairs

**Fig. 3.**
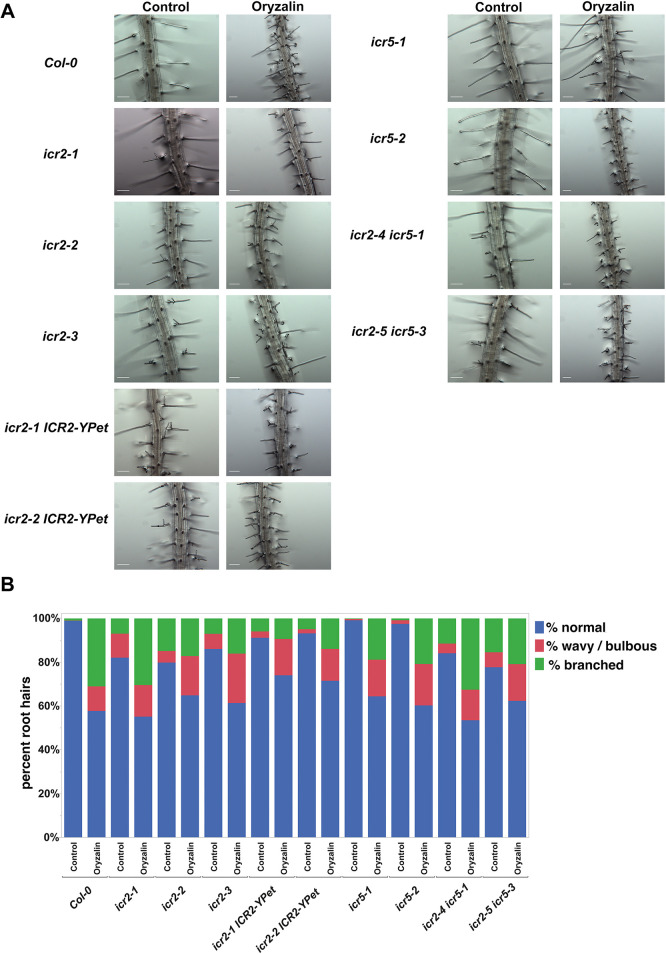
***icr2* mutants develop deformed split root hairs.** (A) Representative images for each genotype with and without 2 days of 200 nM oryzalin treatment. Scale bars: 100 µm. (B) Percentages of morphology types of root hairs in seedlings at 7 DAG. *n*=8 seedlings for each line; 368≥ root hairs per genotype. Experiments were repeated twice. See Table S11 for the data shown in Fig. 3B.

ICR2 expression was detected in root hairs (Fig. S1D). As ROP signaling plays a central role in root hair tip growth (Bloch et al., 2005, 2011; Carol et al., 2005; Chai et al., 2016; Denninger et al., 2019; Duan et al., 2010; Jones et al., 2002; Kang et al., 2017; Molendijk et al., 2001; Nakamura et al., 2018; Wan et al., 2017), we asked whether single, double and triple ICR mutants develop abnormal root hairs. All the plants with *icr2* mutant alleles exhibited altered root hair morphology (Fig. 3A). Quantification of root hair morphology indicated 18%, 20% and 14% of root hairs in the *icr2-1*, *icr2-2* and *icr2-3*, respectively, had either branched or wavy morphology (Fig. 3B). In contrast, *icr5* root hairs were normal with occasional wavy or split root hairs, as also seen in wild-type Col-0, and the *icr2 icr5* double mutants showed no additive effects (Fig. 3A,B). Furthermore, there was partial complementation of the split root hair phenotype in *icr2-1* and *icr2-2* by ICR2-YPet (Fig. 3A). Previously, disrupted root hair growth and formation of split root hairs were observed in mutants of ARMADILLO REPEAT-CONTAINING KINESIN 1 (ARK1), which promotes microtubule destabilization (Eng and Wasteneys, 2014). In the case of *ark1-1* mutant seedlings, the altered root hair growth morphology could be rescued by treatments with low concentrations of the microtubule-destabilizing drug oryzalin (Eng and Wasteneys, 2014). However, when treated with 200 nM oryzalin, both Col-0 and the *icr* mutants responded similarly with around 25-45% split or wavy root hairs (Fig. 3A,B). These data indicate that the function of ICR2 in root hair growth regulation is not redundant with ICR5 and that the mechanisms underlying the aberrant root hair morphology in *icr2* mutants is different from that of the *ark1* mutants. To observe whether mutations in ICR2 and ICR5 have any other effect on root hair development, we measured the distance of the first root hair bulge from the root tip (Fig. S7A; Table S16), the root hair length (Fig. S7B; Table S16) and root hair density (Fig. S7C; Table S16). None of these characteristics differed in the *icr* mutants compared with Col-0 plants. This finding suggested that ICR2 is involved specifically in the polarity maintenance of growing root hairs but not in root hair initiation. Interestingly, the development of split root hairs was also detected in double mutants of ROP and ROPGAP interactors, ARMADILLO REPEAT ONLY (ARO) proteins, which delimit the size of active ROP domains (Kulich et al., 2020).

### Interaction of ICR2, ICR3 and ICR5 with microtubules and ROPs *in vivo*

**Fig. 4.**
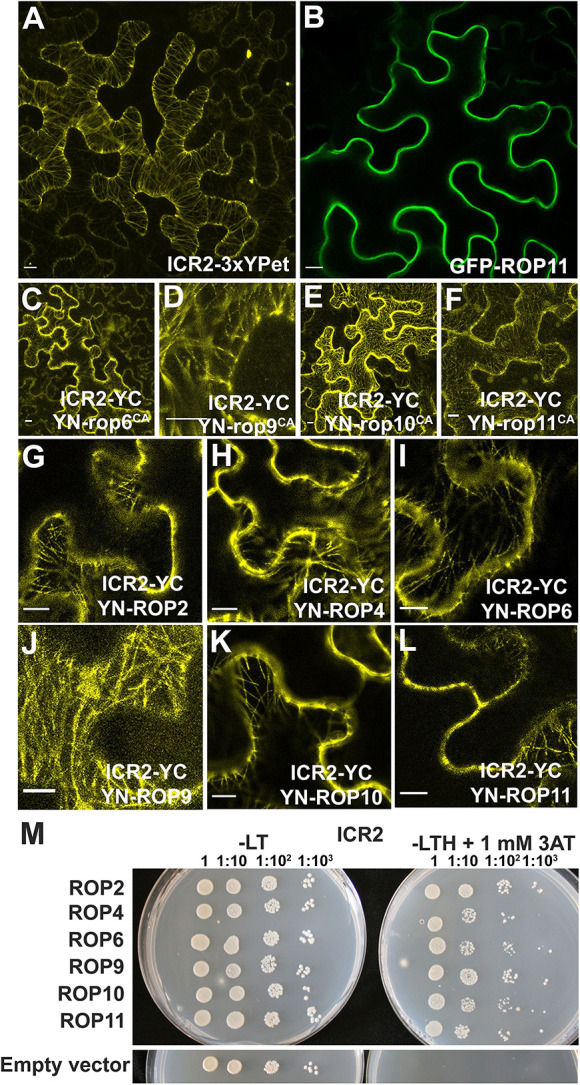
**ICR2 is a microtubule-associated protein that interacts with ROP GTPases.** (A,B) Images of *N. benthamiana* leaf epidermis transiently expressing ICR2-3×YPet or GFP-ROP11. Scale bars: 10 μm. (C-L) BiFC images of *N. benthamiana* leaf epidermis transiently expressing ICR2-YC and (C) YN-rop6^CA^, (D) YN-rop9^CA^, (E) YN-rop10C^A^, (F) YN-rop11^CA^, (G) YN-ROP2, (H) YN-ROP4, (I) YN-ROP6, (J) YN-ROP9, (K) YN-ROP10 and (L) YN-ROP11. Scale bars: 10 μm (C-F); 20 μm (G-L). The YFP signal for panels G-I was separated by linear unmixing. Images for panels A,C-F are *z*-projections of multiple confocal sections. (M) Yeast two-hybrid assays of ICR2 with ROP2, ROP4, ROP6, ROP9, ROP10 and ROP11. −LT, Leu- and Trp-deficient medium; −LTH, Leu-, Trp- and His-deficient medium; 3AT, 3-amino-1,2,4-triazole. Numbers above the panels denote the dilution series. Experiments were repeated twice.

**Fig. 5.**
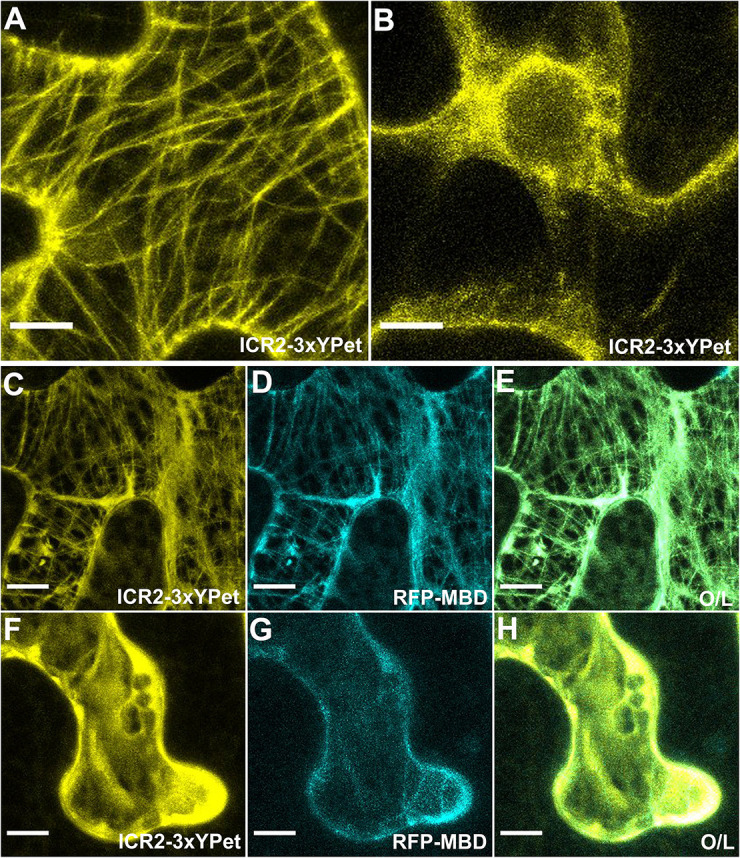
**ICR2 localization with a microtubule marker is disrupted by oryzalin.** (A) Image of *N. benthamiana* leaf epidermis that transiently expresses ICR2-3×YPet. (B) Image of *N. benthamiana* leaf epidermis that transiently expresses ICR2-3×YPet after oryzalin treatment, which disrupts microtubules. (C-E) Images of *N. benthamiana* leaf epidermides that transiently express ICR2-3×YPet and RFP-MBD. (F-H) Imaged of *N. benthamiana* leaf epidermides that transiently express ICR2-3×YPet and RFP-MBD after oryzalin treatment. Images are representative of two repeats. O/L, overlay. Scale bars: 10 µm.

Similar to secondary cell wall pits in the MX, the split root hair phenotype has been associated with perturbation in microtubules and was described for several MAP mutants (Kang et al., 2017; Sakai et al., 2008; Whittington et al., 2001; Yang et al., 2007; Zhang et al., 2015). We discovered ICR2 in a yeast two-hybrid screen with constitutively active ROP10 (rop10C^A^) as bait (Lavy et al., 2007). Transient expression in *Nicotiana benthamiana* leaf epidermis cells showed that, when individually expressed, ICR2, ICR3 and ICR5 localize along cortical microtubules (Fig. 4A, Fig. 5A,C-E; Fig. S8), whereas ROP11 localizes to the plasma membrane (Fig. 4B), as has previously been demonstrated (Bloch et al., 2005; Lavy et al., 2002; Lavy and Yalovsky, 2006). The localization of ICR2 on microtubules was verified by treatment with the microtubule inhibitor oryzalin, which resulted in the disappearance of ICR2-labeled microtubules and a shift of ICR2 to the cytoplasm (Fig. 5B). Similarly, filamentous ICR3 and ICR5 localization disappeared following oryzalin treatments, indicating that they were both localized to microtubules (Fig. S8A-D). Localization of ICR2, ICR3 and ICR5 to microtubules was further verified by co-expression with the microtubule marker RFP-MBD (Fig. 5F-H; Fig. S8E-J).

The interaction of ICR2 with ROPs was examined using BiFC and by yeast two-hybrid assays. For BiFC, ICR2 fused at its C-terminus to the C-terminal half of YFP (ICR2-YC) was transiently expressed in *N. benthamiana* leaf epidermis cells along with various ROPs fused at their N-termini to the N-terminal half of YFP (YN-ROPs). ICR2 interacted with constitutively active versions of ROP6 (type I ROP; Feiguelman et al., 2018) and ROP9, ROP10 and ROP11 (type II ROPs), and with wild-type ROP2, ROP4 and ROP6 (type I ROPs) and wild-type ROP9, ROP10 and ROP11 (type II ROPs). The complexes localized along cortical microtubules (Fig. 4C-L). Similarly, ICR3/ICR5 and ROP11-reconstituted YFP complexes were localized on microtubules (Fig. S8K-L). In yeast two-hybrid assays, ICR2 interacted with both the type I ROPs, ROP2, ROP4 and ROP6, and with the type II ROPs, ROP9, ROP10 and ROP11 (Fig. 4M). Taken together, these results suggest that ICR2, ICR3 and ICR5 are ROP-interacting microtubule-associated proteins.

### ICR2 binds microtubules *in vitro*

ICRs localize to microtubules *in vivo* (Hazak et al., 2019; Mucha et al., 2010; Oda and Fukuda, 2012, 2013; Oda et al., 2010), but it is possible that their localization could have resulted from interaction with a third component rather than direct interaction with microtubules. To examine whether ICR2 is indeed a MAP, we tested its interactions with microtubules *in vitro* using three independent assays.

**Fig. 6.**
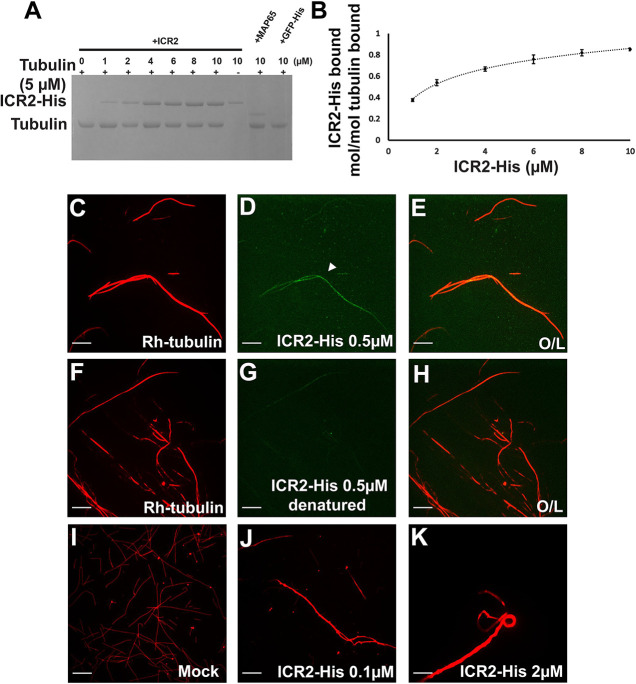
**ICR2 interacts with microtubules *in vitro*.** (A) Coomassie Brilliant Blue-stained SDS-PAGE gel of recombinant ICR2-His_6_ co-sedimented with taxol-stabilized microtubules pre-polymerized from 5 μM tubulin. His-AtMAP65-1 was used as a positive control and GFP-His as a negative control. (B) Quantification of the ICR2-His_6_ band in panel A. The plot averages represent three replicates, error bars represent the s.e.m. (C-H) Immunofluorescence images of rhodamine-labeled tubulin (red) mixed with non-labeled tubulin and polymerized into microtubules in the presence of fluorescein-labeled ICR2-His_6_ (green). Arrowhead in panel D indicates ICR2 on a microtubule. Denatured ICR2-His was used as control. (I-K) Images of rhodamine-labeled tubulin bundling in the presence of 0, 0.1 or 2 µM ICR2. Experiments were repeated three times. O/L, overlay. Scale bars: 10 μm. See Table S12 for the data shown in Fig. 6B.

First, *Escherichia coli*-expressed, affinity-purified ICR2-His_6_ at concentrations ranging between 1 and 10 µM was incubated with preformed taxol-stabilized microtubules. The protein mixtures were precipitated by centrifugation at 100,000 ***g***, and the precipitated proteins were separated by SDS-PAGE and visualized by Coomassie Blue staining (Fig. 6A; Fig. S9A). The levels of precipitated ICR2-His_6_ were quantified by densitometry of the relevant bands, and protein amounts (in moles) were calculated based on the amount of the protein in each reaction and the molecular mass of the proteins (Fig. 6B). MAP65, a known microtubule-interacting protein, was used as a positive control. Based on the densitometry of the bands on the gel, the binding of recombinant ICR2 to microtubules was saturated at stoichiometry of 0.85 mol ICR2-His_6_ per mole of tubulin. This suggests that ICR2 interacts directly with microtubules.

Second, *in vitro* immunofluorescence assays were used to examine whether ICR2 colocalizes with polymerized microtubules. To visualize microtubules, taxol-stabilized microtubules composed of tubulin mixed with rhodamine-labeled tubulin were incubated with ICR2-His_6_. Visualization by *in vitro* immunolocalization established that ICR2 is a MAP (Fig. 6C-E). Incubation with denatured ICR2-His_6_ was used as a negative control (Fig. 6F-H). Some residual colocalization of ICR2-His_6_ was noticed, possibly caused by incomplete denaturation or protein refolding. ICR2 evenly distributed along microtubule filaments.

Third, we carried out an *in vitro* microtubule-bundling assay. To this end, taxol-stabilized microtubules composed of tubulin mixed with rhodamine-labeled tubulin were incubated with increasing concentrations of ICR2. ICR2 at a concentration 0.1 µM was sufficient to cause bundling (Fig. 6I-K; Fig. S9). It is important to note that *in vitro* bundling is not necessarily an indicator for the *in vivo* function of ICR2, rather, bundling *in vitro* is common for many MAPs. Taken together, the *in vitro* precipitation, colocalization and bundling assays confirmed that that ICR2 is a MAP.

### ICR2 colocalizes with microtubules in plants

**Fig. 7.**
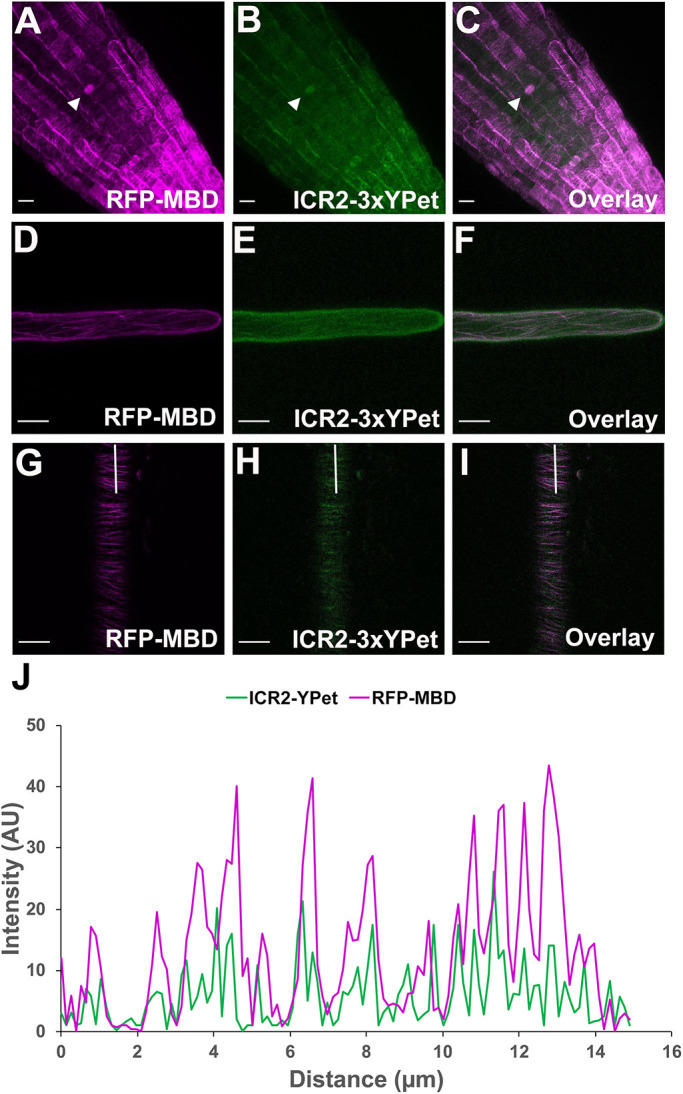
**ICR2-3×YPet colocalizes with microtubules.** (A-C) Images of *icr2-2* roots that express ICR2-3×YPet (green) and RFP-MBD (magenta). ICR2 was detected during interphase at the root tip, in the lateral root cap and in dividing cells in the root cortex, as indicated by arrowheads. (D-F) Images of root hair shank in *icr2-2* plants that express ICR2-3×YPet (green) and RFP-MBD (magenta). (G-I) Images of differentiation/elongation zone epidermis in *icr2-2* plants that express ICR2-3×YPet (green) and RFP-MBD (magenta). Scale bars: 10 µm. (J) Fluorescence intensity profile of RFP-MBD and ICR2-3×YPet signals along the white lines in panels G-I. AU, arbitrary units. See Table S13 for the data shown in Fig. 7J.

**Fig. 8.**
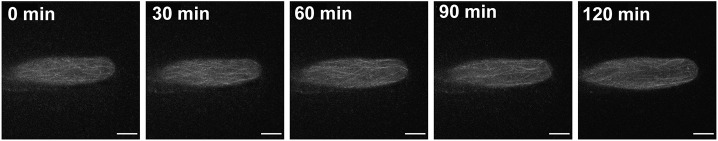
**ICR2-3×YPet localizes to microtubule filaments at the root hair shank during root hair elongation.** Maximum-intensity projection of ten focal planes of *pICR2::ICR2-3×YPet* during time-lapse imaging of growing root hairs. Images were de-noised using Nd-Safir (https://allgo18.inria.fr/apps/ndsafir; Boulanger et al., 2010). Scale bars: 10 µm.

To characterize the subcellular localization of ICR2, we generated a marker composed of the genomic sequence of *ICR2* (including introns) and its promoter fused with the sequence for 3×YPet. To reduce potential steric hindrance, a 33-amino-acid linker was placed between ICR2 and the 3×YPet tag. The inclusion of the linker followed unsuccessful attempts to complement *icr2* phenotypes using GFP-tagged ICR2 without a linker. To avoid potential mis-localization due to overexpression, the *pICR2::ICR2_genomic_:3×YPet* construct was transformed into two *icr2* T-DNA insertion mutants, *icr2-1* and *icr2-2*, that also express the microtubule marker RFP-MBD (*icr2-1×UBQ10::RFP-MBD* and *icr2-2×UBQ10::RFP-MBD*). Importantly, the *pICR2::ICR2_genomic_:3×YPet* fusion partially complemented the *icr2-1* and *icr2-2* pit formation and root hairs phenotypes, confirming its functionality (Figs 1–3). In the lateral root cap, root hairs and root epidermis cells, the ICR2-3×YPet fusion protein was observed on cortical microtubules (Fig. 7A-I). The localization of ICR2 on microtubules was confirmed by colocalization with the microtubule marker RFP-MBD (Fig. 7C,F,I,J). In growing root hairs, ICR2-3×YPet was observed on microtubules at the root hair shank (Fig. 8; Movie 1). The localization of ICR2 on microtubules in root hairs suggested that the split root hair phenotype of the *icr2* mutants is associated with ICR2 function on microtubules. Given the similarities between the phenotypes of the *icr2* loss-of function and ROP2 gain-of-function mutants (Jones et al., 2002; Kang et al., 2017), it is possible that ICR2 is not a ROP2 effector but may function as a MAP independently, or possibly as a ROP10 effector at root hair shanks (Hirano et al., 2018), or may interact with ROP2 in some other manner.

In dividing cells, ICR2 was colocalized with microtubules during mitosis (Fig. S10A). ICR2-3×YPet colocalized with RFP-MBD in all mitotic stages including the preprophase band, the spindle during metaphase and anaphase, and the expanding phragmoplast microtubules in telophase (Fig. S10B). ICR2 localization on microtubules during cell division matched the co-expression data, which showed high correlations with cell division and cytoskeleton genes (Table S1). Yet, neither the *icr2* single mutants nor the *icr2 icr5* double mutants displayed abnormal cell division phenotypes. Thus, the function of ICR2 is likely redundant with other MAPs during cell division and remains to be explored.

**Fig. 9.**
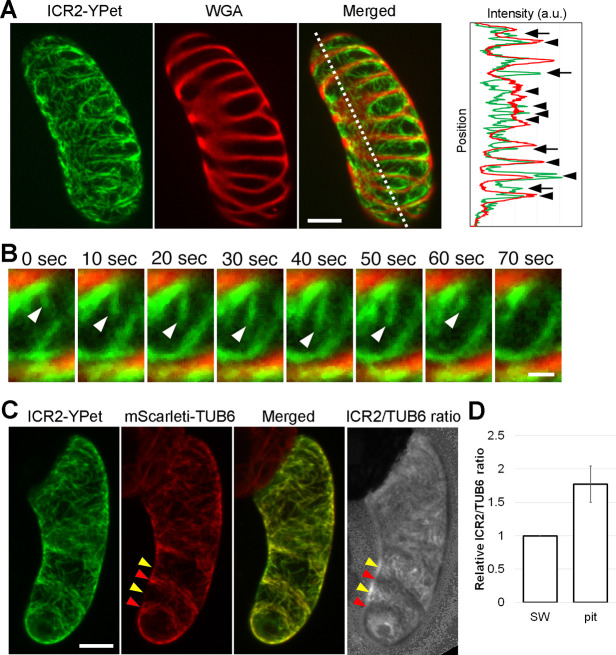
**ICR2 in VND6-induced xylem vessel cells.** (A) Maximum-intensity projection of ICR2 (*pLexA:ICR2-YPet*) and secondary cell walls stained with WGA-Alexa Fluor 561. The plot shows the intensity profile along the dotted line. Arrowheads and arrows indicate ICR2 signals at areas outside and inside of pits, respectively. a.u., arbitrary units. (B) Ten second interval time-lapse images of ICR2 and WGA. White arrowheads indicate ICR2 associating to growing microtubules. (C) ICR2 (*pLexA:ICR2-YPet*) and microtubules (*35S:mScarleti-TUB6*) in VND6-induced xylem vessel cells. Yellow and red arrowheads indicate microtubule-sparse (pit) and microtubule-rich areas, respectively. Note that the ICR2/TUB6 signal ratio in microtubule-depleted areas is higher than that in microtubule-rich areas. (D) Relative ICR2/TUB6 signal ratio on microtubules at microtubule-rich (SW) and -sparse (pit) areas. Data are mean±s.d. (*n*=8 cells, 16 to 32 microtubules were analyzed in each cell). Scale bars: 10 μm (A,C); 2 μm (B). See Table S14 for the data shown in Fig. 9A,D.

The pit phenotype of *icr2* mutants prompted us to examine ICR2 localization in vascular tissues. Unfortunately, the experimental setup available to us for imaging of microtubules in the vasculature and the low expression levels of ICR2 made direct imaging impossible. To overcome this difficulty, we used an *in vitro* xylem differentiation system to view ICR2 in VND6-induced xylem vessel cells. Expressing *pLexA:ICR2-YPet* enabled better localization analysis in these cells, both of ICR2 with the secondary cell wall and with microtubules. ICR2-YPet was transformed into 7-day-old suspension cells harboring *LexA:VND6*, before inducing xylem differentiation. The cells were then imaged after seven additional days. ICR2 localized both in and out of differentiating pits (Fig. 9A). Furthermore, ICR2-YPet associated with growing microtubules (Fig. 9B). ICR2-YPet colocalized with mScarleti-TUB6, in both microtubule-rich areas under the secondary cell wall and microtubule-sparse areas in the pit area (Fig. 9C). The ICR2/TUB6 ratio in the microtubule-sparse pit area was higher than in the microtubule-rich area of the secondary cell wall (Fig. 9D). ICR2 colocalization with microtubules in differentiating xylem cells and the pit phenotype of *icr2* mutants, taken together with the previously published data on the interaction of ICR2 with KINESIN-13A (Mucha et al., 2010) and the mechanism of ICR5 function in pit formation (Oda and Fukuda, 2012), suggest that the association of ICR2 with microtubules is crucial for its function in pit patterning.

### *icr2* mutants display altered microtubule organization and dynamics

**Fig. 10.**
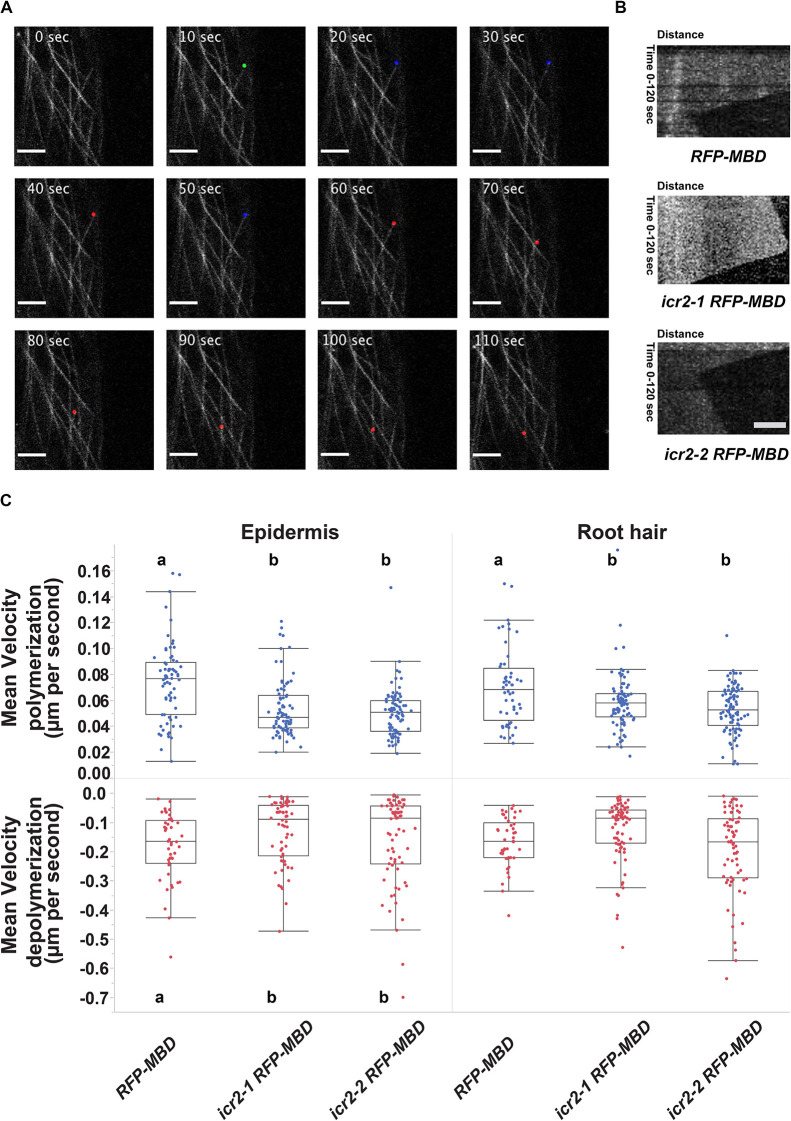
**ICR2 affects microtubule dynamics in the root epidermis and root hairs.** (A) Representative time-lapse imaging of RFP-MBD-labeled microtubules. Images were de-noised using Nd-Safir (https://allgo18.inria.fr/apps/ndsafir; Boulanger et al., 2010). Filament extension, pause and shrinkage are labeled with green, blue and red dots, respectively. Scale bars: 5 µm. (B) Kymographs tracking RFP-MBD-labeled microtubule tips in Col-0, *icr2-1* and *icr2-2* seedlings. Scale bar: 5 μm. (C) Quantification of microtubule extension and shrinkage rates. Means with different letters are significantly different (Tukey's HSD, *P*<0.05). One-way ANOVA results are in Table S3. The boxes are the interquartile ranges, the whiskers represent the first and fourth quartiles, and the lines are the averages. *n*>77 filaments per genotype. The imaging was carried out in multiple sessions. See Table S15 for the data shown in Fig. 10.

The localization of ICR2 to microtubules as well as its *in vitro* binding to microtubules suggests that it may affect the organization and dynamics of microtubules. To test this, *icr2-1* and *icr2-2* plants were crossed with *UBQ10::RFP-MBD*, and analysis of microtubule dynamics was carried out on non-segregating double homozygous plants using high-frequency time-lapse imaging and tracking of individual microtubule filaments. The tracking data (Fig. 10A) were used to create kymographs (Fig. 10B), which were then used to calculate microtubule growth and shrinkage rates, the time spent in each condition, transition times and pauses in growth/shrinkage. In root epidermal cells as well as root hairs, microtubule growth rates were significantly slower in the *icr2* mutants than in Col-0 plants (*P*>0.001) (Fig. 10C). In contrast, shrinkage rates were lower only in the epidermis (Fig. 10C). Additionally, time spent at pause was higher in mutant root epidermal cells than in those of Col-0 plants (Fig. S11), and the transitions between filament growth, shrinkage and pause occurred at higher frequency in the *icr2* mutants than in Col-0 plants (Fig. S12).

The analysis of microtubule dynamics indicated that in addition to transduction of ROP signaling, ICR2 may regulate root hair growth by affecting microtubule stability. Yet, as previously shown in Fig. 3, the mechanism is likely different from other MAP mutants such as *ark1-1* (Eng and Wasteneys, 2014). The analysis further showed that ICR2 regulates microtubule dynamics differently in different cell types. This cell-type-specific regulation of microtubule dynamics by ICR2 is consistent with the functional divergence of *icr2* and *icr5* developmental phenotypes. Abnormal root hair growth in *icr2* mutants but not in the *icr5* single mutant (Fig. 3), and, likewise, the secondary cell wall deposition phenotype of *icr5* but not of *icr2* in the PX (Fig. 2) support cell- or tissue-specific functions of ICR2 and ICR5.

## DISCUSSION

### The functions of ICR2 and ICR5

ICR proteins have been suggested to function as adaptors that mediate the interaction of ROPs with distinct target proteins (Lavy et al., 2007; Li et al., 2020; Mucha et al., 2010; Oda and Fukuda, 2012). The combined phenotypic analysis of the mutants and the subcellular localization analysis presented in this work indicate that the function of the ICRs is more complex than previously thought. The results of our work as show that ICR2 is a MAP, which stably associates with microtubules in different cell types and during all phases of the cell cycle, affecting microtubule dynamics. Through its interaction with activated ROPs, ICR2 links ROPs and microtubules. This interaction may result in localized microtubule reorganization and destabilization as has previously been demonstrated for ICR5 and ICR1 (Hazak et al., 2019; Mucha et al., 2010; Oda and Fukuda, 2012, 2013; Oda et al., 2010; Sternberg et al., 2021). In addition, the increased pit density in the *icr2* and *icr5* single mutants and in the *icr2 icr5* double mutants, as well as the split root hair phenotype of the *icr2* mutants, may suggest that ICR2 and likely ICR5 can also restrict ROP signaling. Furthermore, the phenotypic analysis showed that the function of ICR2 and ICR5 is partially cell specific, suggesting some functional diversification of these ICRs, and that ICR2 has ROP-independent functions in the root hairs that affect microtubule dynamics.

### ICR2 is a MAP

The *in vitro* and *in vivo* analyses unequivocally demonstrate that ICR2 is a MAP that stably associates with microtubules in all the different cells and tissues tested throughout the cell cycle during interphase and cell division. ICR2 was also observed on microtubules when it was ectopically overexpressed under the *35S* promoter either alone or with ROPs (Figs 4,5). Collectively, these data indicate that for analyzing ICR2 function, it is crucial to use the full-length protein and that analysis of truncated versions that lack the microtubule-binding domain would lead to erroneous results. Importantly, our data indicate that ICR3 and ICR5 are also ROP-interacting MAPs.

### The involvement of ICR2, ICR5 and ICR3 in secondary cell wall patterning

ROP11 was previously implicated in the regulation of secondary cell wall pits. A model involving ICR5 in the process was suggested previously (Oda and Fukuda, 2012; Oda et al., 2010). In this model, locally activated ROP11 recruits ICR5, leading to depolymerization of cortical microtubules in the future pit regions. Here, we found that *icr2* and *icr5* single mutants as well as *icr2 icr5* double mutants have significantly smaller, yet denser pits compared with those of Col-0. Interestingly, pit sizes are not significantly different between the *icr2* and *icr5* single and double mutants. These data indicate that ICR2 and ICR5 have common functions in MX pit formations. The partial complementation of pit size and density in *icr2-1* and *icr2-2* by ICR2-3×YPet and the localization of ICR2-3×YPet on microtubules in trans-dedifferentiating tracheary elements, which is similar to that of ICR5 (Oda and Fukuda, 2012; Oda et al., 2010), further support the function of ICR2 in addition to that of ICR5 in pit formation. Importantly, the smaller average pit sizes in the *icr2* and *icr5* single and double mutants are only partially due to decreased maximal pit size and also to the formation of very small pits.

The formation of smaller pits is consistent with the previously proposed function of ICR5. Yet, the increased pit densities indicate that the mechanism of ICR2 and ICR5 function is more complex. Hence, on the one hand, KINESIN-13A-dependent microtubule destabilization is reduced in the *icr2* and *icr5* single and double mutants, leading to smaller pits. This role of ICR2 is supported by the distribution of ICR2-3×YPet in trans-differentiating tracheary elements between secondary cell wall-enriched and -free areas, and the higher ICR2-3×YPet/tubulin ratio in the secondary cell wall-free areas. On the other hand, additional ROP nanodomains are formed in the plasma membrane, leading to increased pit formation in both *icr2* and *icr5* single and double mutants. In BiFC assays, both ICR2 and ICR5 recruited ROP11 to microtubules, suggesting that they could restrict the formation of ROP domains. Further studies will be required to elucidate the ROP domain-ICR-microtubule interaction. The complementation of pit density and partial complementation of pit size in *icr2-1* and *icr2-2* by ICR2-3×YPet implicate ICR2 in the regulation of pit size and indicate that its levels are crucial for the maintenance of pit density. Furthermore, the microtubule dynamics analysis showed decreased microtubule growth rates in the *icr2* mutant background, indicating that ICR2 could affect microtubule stability and in turn pit formation by additional mechanisms.

The higher density of secondary cell wall coils in the PX of *icr5* is in line with a role of ICR5 in the destabilization of microtubules and possibly modulation of ROP domain sizes. Based on a combination of experimental work and computer simulation, Schneider et al. (2021) recently proposed that microtubule destabilization takes place during PX secondary cell wall formation. It is possible that ICR5 functions during this microtubule destabilization, but further studies on microtubule dynamics in *icr5* mutants will be required to elucidate its function.

Using BiFC and yeast two-hybrid assays, we detected neither homodimerization nor heterodimerization of ICR2, ICR3 or ICR5 (Figs S5 and S6). These protein interaction data suggest that ICR2, ICR3 and ICR5 do not function together in a complex. Publicly available transcriptomics data (https://bioit3.irc.ugent.be/plant-sc-atlas/; Graeff et al., 2021) suggest that their expression patterns may partially explain the overlap as well as cell-type-specific functions of *ICR2* and *ICR5*. Both ICR2 and ICR5 are expressed in the PX, and the expression of ICR5 is significantly higher, making it the predominant ICR in this tissue. The differences in expression in the MX between the two ICRs are less extensive and, thus, loss of function of each ICR contributes to the pit formation phenotype.

### The function of ICR2 in root hair growth

The split root hair phenotype of the *icr2* mutant is not associated with changes in root hair density or the position of the trichoblasts. This indicates that ICR2 function is required for the maintenance of polar root hair elongation. The localization of ICR2 on microtubules in growing root hairs and the altered microtubule dynamics of *icr2* mutants (i.e. slower microtubule growth rate and increased rate of transitions between filament extension, pause and shrinkage) indicate that ICR2 is necessary for the stability of microtubules in root hairs. A split root hair phenotype has been associated with perturbation of microtubules and was described for several MAP mutants, which has been attributed to effects on microtubule stability (Eng and Wasteneys, 2014; Sakai et al., 2008; Whittington et al., 2001) or on ROP2 function (Kang et al., 2017; Yang et al., 2007; Zhang et al., 2015). ICR2 is a ROP-interacting protein and a MAP, and affects microtubule stability. Hence, it may affect root hair polar growth by either interacting with ROP or affecting microtubule stability. Interestingly, unlike in the *ark1* mutants (Eng and Wasteneys, 2014), the split root hair phenotype of *icr2* mutants could not be rescued by treatments with low concentration of oryzalin (Fig. 3), suggesting that ICR2 and ARK1 likely do not affect root hair polar growth through similar mechanisms.

In root hairs, ICR2 was found to localize on microtubules along the shank and not in ROP2 domains at the root hair tip (Jones et al., 2002). Furthermore, similar to the *icr2* mutant phenotype, ROP2 gain-of-function mutations and reduced ROP2 inactivation in the *aro* mutants led to the formation of split root hairs (Jones et al., 2002; Kang et al., 2017; Kulich et al., 2020). ICR2 interacted with ROP2 in yeast two-hybrid and BiFC assays (Fig. 4). It is possible that in root hairs, ICR2 interaction with ROP2 results in the recruitment of ROP2 away from its active domain to the microtubules. Additionally, ROP10 was shown to regulate cell wall formation at the shank, leading to root hair shank hardening (Hirano et al., 2018). Taken together, these data suggest that ICR2 may function as a ROP10 effector in root hairs or may have a ROP-independent function.

A recent study implicated ICR2 in the recruitment of the protein kinase AGC1.5 to root hair tips, where it was proposed to phosphorylate ROPGEF4 and ROPGEF10 to promote root hair growth (Li et al., 2020). However, the altered root hair phenotype of the *icr2* mutants and the localization of ICR2 on microtubules in root hairs are not compatible with the proposed function of ICR2 in the activation of ROP2 function via AGC1.5 and ROPGEF4/10. Importantly, the distribution of ICR2 reported by Li et al. (2020) was determined by the analysis of ICR2 with N-terminally tagged fluorescent proteins, which likely disrupted the interaction of ICR2 with microtubules as it takes place via the N-terminal end of ICR2. As a result, ICR2 was observed at the plasma membrane in root hairs or when co-expressed with ROP2, whereas its colocalization of microtubules was not observed. Hence, although the interaction of ICR2 with AGC1.5 is intriguing, its functional role will require additional investigation.

The results of this study, in combination with earlier works, suggest that the ICR family proteins have multiple unique roles as MAPs involved in microtubule dynamics and as ROP effectors. ICR2 and ICR5 may affect microtubule destabilization through their interactions with proteins such as KINESIN-13A. In contrast, the analysis of microtubule dynamics indicates that ICR2, as well as ICR5, is involved in microtubule organization and dynamics, and mediates ROP signaling to microtubules directly or through yet unknown target proteins. The tissue- and cell-type-specific functions of ICR2 and ICR5 may reflect interactions with different proteins in different cells, as well as cell-type-specific expression. Although ICR2 localized to microtubules in interphase as well as during cell division, we did not detect any cell division abnormalities in the *icr2* single mutants or in the *icr2 icr5* double mutants. The function of ICR2 during cell division will be the focus of future studies.

## MATERIALS AND METHODS

### Molecular procedures

#### Plasmid DNA purification

Plasmid purification was carried out with a DNA-spin Plasmid DNA Purification Kit (iNtRON Biotechnology) according to the manufacturer's protocol.

#### PCR

PCR was used for gene detection and cloning. For general uses such as colony screening, Taq DNA polymerase (Fermentas) was used. To eliminate error, for cloning purposes, PCR reactions were carried with the proof-reading Phusion DNA polymerase (New England Biolabs). Reaction conditions were used based on the enzyme manufacturer's instructions, with annealing temperatures chosen based on primers. All oligonucleotide primers used in this study are listed in Tables S4 and S5.

#### DNA fragment extraction from agarose gels

DNA extraction from agarose gels was done using the Gel extraction kit QIAEX II (QIAGEN).

#### Cloning for yeast two-hybrid assay, transient expression and BiFC assay

To obtain constitutively active ROP mutants, the respective genes were mutated by site-directed mutagenesis. The mutation Q67L was introduced in ROP6 and ROP10, and G15V was introduced in ROP9 and ROP11. For yeast two-hybrid analysis, the ROP2, ROP4, ROP6, ROP9, ROP10 and ROP11 coding sequences were subcloned into *pGBT9.BS* (Clontech). The ICR2, ICR3 and ICR5 coding sequences were cloned into *pGAD.GH* (Clontech) and *pGBT9.BS* (Clontech). For BiFC assays, YN-ROP2, YN-ROP4, YN-ROP6, YN-ROP9, YN-ROP10, YN-ROP11 and ICR2-YC sequences were subcloned into *pB7m34GW* (Karimi et al., 2005) by the Three-Way Gateway standard protocol (Thermo Fisher Scientific). The expression cassette included the CaMV *35S* promoter, tag, gene of interest, and *NOS* terminator. ICR3-GFP, ICR5-GFP, ICR2-YN, ICR3-YN, ICR3-YC, ICR5-YN and ICR5-YC were cloned by restriction-digestion using the GreenGate cloning system (Lampropoulos et al., 2013). The expression cassette included the CaMV *35S* promoter, gene of interest, tag and the *UBQ10* terminator.

#### Creation of pB7-pICR2::ICR2

Intermediate vectors were created using Gateway BP Clonase (Table S8). A 2493-bp fragment harboring the entire genomic sequence of *ICR2* (AT2G37080) from the ATG initiation codon through the stop codon was amplified from genomic DNA and subcloned into *pDONR221* (Thermo Fisher Scientific). The promoter sequence of *ICR2* (2225 bp upstream of the *ICR2* initiation codon) was likewise amplified and subcloned into *pDONR-P4R1* (Thermo Fisher Scientific). The *NOS* terminator was subcloned into *pDONR-P2R3* (Thermo Fisher Scientific). All three intermediate vectors were further cloned into the *pB7m34GW* destination vector using the Gateway LR Clonase II Plus Enzyme Mix (Table S8) for MultiSite LR recombination reaction.

#### Creation of *pK7-pICR2::ICR2-3×YPet*

Intermediate vectors were created using Gateway BP Clonase. A 2490-bp fragment harboring the entire *ICR2* (AT2G37080) genomic sequence from the ATG initiation codon but without the final stop codon was amplified from genomic DNA and subcloned into *pDONR221*. The promoter sequence of *ICR2* (2225 bp upstream of the *ICR2* initiation codon) was likewise amplified and subcloned into *pDONR-P4R*. 3×YPet-3×HA (Marquès-Bueno et al, 2015) was received from NASC (N2106295). This vector contains a 33-amino acid linker (DPAFLYKVARLEEFGTPGSKSISLDPLPAAAAA) between ICR2 and the three repeats of the fluorescent protein YPet to reduce potential steric hindrance. All three intermediate vectors were further cloned into *pK7m34GW* using the Gateway LR Clonase II Plus Enzyme Mix for MultiSite LR recombination reaction.

#### Creation of ICR2-His_6_

*pET28b-ICR2-His_6_* was created by amplifying a 1749-bp fragment of the coding sequence of *ICR2* without the stop codon and subcloning into *pJET1.2* (Thermo Fisher Scientific). It was than integrated into *pET28b* (EMD Biosciences) using primers containing overlapping sequences with *pET28b* and amplifying the entire vector by Transfer PCR (Erijman et al., 2013).

#### Multiplex genome-editing design and constructs

The polycistronic tRNA-gRNA system was used to generate multiple short guide RNAs (sgRNAs) with different target sequences by flanking the sgRNAs with a tRNA precursor sequence as previously described (Xie et al., 2015). See supplementary Materials and Methods for further details.

#### Sequencing

DNA sequencing was performed at the Tel Aviv University DNA sequencing facility and was carried using the BigDye Terminator Cycle Sequencing Kit (Applied Biosystems).

### Plant genomic DNA isolation

Typically, 100 mg of liquid N_2_ batch-frozen leaf tissue was ground with a mortar and pestle, and genomic DNA was isolated using the GenElute Plant Genomic DNA Kit (Sigma-Aldrich), according to the manufacturer's protocol.

### Total RNA isolation from plants

*Arabidopsis* seedlings were batch frozen using liquid N_2_, and tissue was ground with a mortar and pestle. Total RNA was isolated from the ground material using the RNeasy SV total RNA isolation kit (QIAGEN) according to the manufacturer's instructions.

### RT-PCR

cDNA synthesis for standard RT-PCR experiments was carried out using the High-Capacity cDNA Reverse Transcription Kit (Applied Biosystems). Approximately 1 µg RNA dissolved in 10 µl H_2_O was added to 10 µl of the 2× Reverse Transcription Master Mix, containing 2 µl 10× RT buffer, 0.8 µl 25× dNTP mix, 2 µl 10× RT random primers, 1 µl MultiScribe reverse transcriptase, 1 µl RNase inhibitor and 3.2 µl nuclease-free H_2_O. The reaction was performed in a thermal cycler for 10 min at 25°C, then for 120 min at 37°C, and 5 min at 85°C (for inactivation).

### Sequence analysis

Sequence analysis was carried out using the SnapGene (GSL Biotech; available at https://www.snapgene.com) sequence analysis software package. The BLAST algorithm (http://www.ncbi.nlm.nih.gov/BLAST) was used to search the DNA and protein database for similarity. Multiple sequence analysis was done using JALVIEW (Waterhouse et al., 2009) with the Clustal (Thompson et al., 1994) algorithm.

### Bacterial strains and growth conditions

*E. coli* DH5α(F′)-*F′* was used for heat shock transformation and molecular cloning. *Agrobacterium tumefaciens* strain *GV3101/pMP90* was used for transient and stable expression of recombinant genes in *N. benthamiana* and *Arabidopsis* as previously described (Lavy et al., 2002). Growth medium for bacteria was prepared as previously described (Lavy et al., 2002). For solid medium, 1.5% w/v of agar was added to the medium. *E. coli* cells were selected on 100 μg/ml ampicillin or 50 μg/ml kanamycin. *Agrobacterium tumefaciens GV3101/pMP90* was selected on 100 μg/ml gentamycin and 50 μg/ml spectinomycin.

### Yeast two-hybrid assays

The *Saccharomyces cerevisiae* strain *PJ69-4a* was used as host. Plasmids for expression of ROPs (*pGBT ROPs*) were co-transformed with *pGAD-ICR2* (Table S6) into yeast cells via a standard lithium acetate transformation protocol. Four decimal dilutions of colonies expressing both plasmids were grown on a medium lacking leucine (L), tryptophan (T) and histidine (H) supplemented with 1 mM 3-amino-1,2,4-triazole (3AT) for interaction detection or on a medium lacking leucine and tryptophan for growth monitoring. The plates were incubated at 28°C.

### Expression of ICR2-His_6_ in *E. coli*

ICR2-His_6_ was transformed into the BL21 (Rosetta) *E. coli* strain. Cells were grown at 37°C to an OD_600_ of 0.5-0.7 and then induced with 1 mM isopropyl-beta-D-thiogalactopyranoside overnight at 16°C. Immediately after induction, cells were harvested by centrifugation at 5000 ***g*** for 15 min at 4°C, and stored at −80°C until further use.

### Purification of ICR2-His_6_

Protein purification was carried out with the AKTA Prime protein purification system (GE Healthcare). First, cells were homogenized by sonication using the VCX500 ultrasonic processor (Sonics & Materials) in washing buffer (50 mM NaH_2_PO_4_, 300 mM NaCl, 20 mM imidazol and 5% glycerol, pH 8.0) containing 1 mM dithiothreitol. ICR1-His_6_ and ICR2-His_6_ recombinant proteins were purified over a His-TRAP FF column (GE Healthcare) with a 1-ml bed volume. The column was washed with 30 ml of washing buffer. The proteins were released with imidazole Elution buffer (50 mM NaH_2_PO_4_, 300 mM NaCl, 250 mM imidazole and 5% glycerol, pH 8.0). The proteins were concentrated with Amicon Ultra-15 filters (Millipore), with molecular weight cutoffs of 50 kDa for ICR2-His_6_, at 4000 ***g*** and 4°C to a final volume of approximately 500 μl. The concentrated protein samples were filtrated through Millex 0.22 μm syringe filters (Millipore), loaded onto a Superdex 200 HR 10/30 gel filtration column (GE Healthcare) and eluted with a gel filtration column buffer (50 mM NaH_2_PO_4_, pH 7.0). To concentrate the protein, an Amicon Ultra-15 centrifugal filter device was used, the protein was centrifuged at 4000 ***g***, and the buffer was exchanged to PEM buffer (0.1 M PIPES, 1 mM EGTA and 1 mM MgCl_2_, pH 6.9). The purified proteins were again concentrated using the Amicon Ultra-15 filters, divided into aliquots, batch frozen in liquid nitrogen, and kept at −80°C until further use. Protein concentrations were determined using the BCA Protein Assay kit (Pierce) according to the manufacturer's protocol.

### Plant materials and transformation

#### Plant materials

*Arabidopsis* Col-0 ecotype was used as wild type in all experiments and was used for all transformations for the generation of transgenic plants. *Nicotiana benthamiana* was used for transient expression in leaf epidermal cells. The *icr2-1* (*GK567F02*), *icr2-2* (*GK281B01*), and *icr2-3* (*GK159B08*) T-DNA mutants were obtained from NASC and are in the Col-0 background. For generating the CRISPR/Cas9-mediated genome-edited mutants, transgenic plants were created by expression of appropriate gRNAs in a single transcriptional unit, spaced by tRNAs under the control of the *AtU6* promoter as described (Xie et al., 2015). The Cas9 in this system was expressed under the control of the *GEX1* egg-specific promoter, and therefore the genomic editing events identified were heritable and not somatic, thus improving the screening process. For analysis, the T-DNA containing the *pGEX1::Cas9-AtU6::tRNA-gRNA* expression cassette was crossed out from all mutants. Seeds for *UBN::RFP-MBD* were a gift from Dr Sabine Müller, University of Tübingen, Germany, and were previously described (Lipka et al., 2014). Plants used and generated in this work are listed in Table S7.

#### Plant growth conditions

Seeds of wild-type Col-0 and transgenic and mutant *Arabidopsis* plants were sown on the soil (Weizmann Institute mix, Pele Shacham Ltd, Ashkelon, Israel) and moved to stratification at 4°C for 48 h in the dark in order to increase the uniformity of germination. The seeds were then moved to a growth chamber under long-day conditions (16 h light/8 h dark, light intensity 100 µE/m^2^s) at ∼22°C. *N. benthamiana* plants were grown in 10 cm pots. Seeds were sown on a mixture of 70% soil with vermiculite (Avi Saddeh mix, Pecka Hipper Gan). Plants were grown in an environmental growth chamber under conditions of long days (16 h light/8 h dark, light intensity 100 µE/m^2^s) at ∼25°C. For growth of *Arabidopsis* on plates, plates contained 0.5× Murashige and Skoog (MS) medium (Duchefa Biochemie), titrated to pH 5.7 with MES buffer, KOH and 0.8% plant agar (Duchefa Biochemie). In some cases, the medium was prepared with 1% sucrose. The seeds were then moved to a growth chamber and placed vertically in most cases, or horizontally for germination assays to grow under long-day conditions (16 h light/8 h dark, light intensity 100 µE/m^2^s) at ∼22°C. Prior to the transfer to the growth chamber, the sown seeds were stratified at 4°C for 48 h in darkness. In both cases, seeds were surface sterilized by evaporation of HCl (6 ml) in sodium hypochlorite (100 ml) in a closed container for 1 h.

#### Stable transformation in *Arabidopsis*

Transformation was performed by the floral dip method as described previously (Clough and Bent, 1998).

#### Transient expression in *N. benthamiana*

Transient expression was carried out as previously described (Lavy et al., 2007).

#### VND6-induced xylem cell differentiation in *Arabidopsis* cell cultures

Induction of metaxylem differentiation in cultured cells was performed as described previously (Oda et al., 2010). Briefly, 1 ml of 7-day-old suspension cells harboring *LexA:VND6* was transferred into a 15-ml tube and diluted with 9 ml of MS medium without 2,4-dichlorophenoxyacetic acid. Cells were allowed to settle for 5 min, after which the upper 5 ml of the medium was removed to adjust cell density. The suspension culture was supplied with 2 μM estradiol (10 mM stock in DMSO, Fujifilm Wako) and 2 μM brassinolide (10 mM stock in DMSO, Fujifilm Wako), and cultured for 24 h. Transformation was performed as described previously (Oda et al., 2010). Seven-day-old cells were co-cultured with the *Agrobacterium tumefaciens* strain GV3101 MP90 (A gift from Dr Csaba Koncz, Max Plant Institute for Plant Breeding Research, Cologne, Germany) harboring LexA:ICR2-1×YPet and 35S:mScarleti-TUB6 for 48 h in MS medium supplemented with 50 mg/l of acetosyringone (Sigma-Aldrich). Claforan (0.5 mg/l; Aventis) was added to the culture, and the suspension cells were cultured for a further 5 days. Cell walls were labeled with WGA-Alexa Fluor 561 (Thermo Fisher Scientific).

### Analysis of microtubule dynamics

Microtubule dynamics were analyzed by high-frequency time-lapse imaging of seedlings of *RFP-MBD*, *icr2-1×RFP-MBD*, and *icr2-2×RFP-MBD* genotypes at 8 DAG. Seedlings were grown on CellView 35/10 mm glass-bottomed cell culture dishes (Greiner, 627860) at a 45° angle, so that roots grew along the glass bottom between the growth medium and the glass. Imaging of root hairs and adjacent root epidermis cells was done at 2-s intervals for a total of 60 frames using an LSM 780-NLO confocal laser scanning microscope (Zeiss) in fast-scanning mode with a 63× water immersion objective, and they were visualized by excitation with an argon laser at 561 nm and spectral GaAsP detector set between 570 nm and 695 nm. Images were de-noised using Nd-Safir (https://allgo18.inria.fr/apps/ndsafir; Boulanger et al., 2010). Quantification of microtubule dynamics was done by tracking individual microtubule filaments. The tracking data were used to create kymographs, which were then used to calculate microtubule growth and shrinkage rates, the time spent at each condition, as well as the transitions between them and pauses in growth/shrinkage. This analysis of imaging data was performed using the KymoToolBox ImageJ plugin (Zala et al., 2013). Typically, five to ten microtubule filaments were analyzed per cell and five cells, each from a different plant, were analyzed for each genotype and cell type. Overall, the number microtubule filaments analyzed was between 77 and 113.

### Secondary cell wall pit area and pit density per area

Analysis of secondary cell wall of the MX pits was carried out on seedling roots at 8 DAG. Roots were imaged using differential interference contrast (DIC) light microscopy after clearing with chloral hydrate:lactic acid (2:1) for 3 days. To quantify the area of secondary cell wall pits, pits were manually selected in DIC images and analyzed using ImageJ. Pit density was calculated as the number of secondary cell wall pits divided by the area of MX vessel cells and expressed as the number of pits per 1000 mm^2^. Two or three cells were imaged for each plant, and four or five plants were analyzed for each genotype.

### Protoxylem lignification

Roots at 7 DAG were imaged for lignin autofluorescence by excitation at 405 nm. Emission was detected with a spectral detector set between 410 nm and 524 nm. *Z*-stacks were taken of six to ten focal planes, and maximum-intensity images were created. Analysis was carried out in the maturation zone of the root on maturing PX cells, which have a well-defined spiral pattern at this region. No MX differentiation was detected. The mean distance between lignified spirals was measured using the semi-automated Cell-o-Tape macro for ImageJ (Fiji). Representative images were taken using DIC microscopy. Five roots were analyzed for each genotype, and in each plant, two PX cells were imaged and quantified.

### Analysis of root hair morphology

Seedlings were initially grown on 0.5× MS agar medium with 1% sucrose for 5 days, then transferred to 0.5× MS agar medium containing 1% sucrose and 200 nM oryzalin for 2 days. Seedlings for each genotype were compared, and the frequency distribution of the morphology types of root hairs was scored.

### Root hair measurements

Root hairs in seedlings at 7 DAG were imaged and measured as previously described (Denninger et al., 2019). The first visible swelling of the cell outline was defined as the first bulge, and the distance to root tip was measured. Root hair density was analyzed in the next 2 mm. Root hair length was measured in a region 3-6 mm away from the root tip.

### Analysis of ICR2-3×YPet in growing root hairs

Microtubule dynamics were analyzed by high-frequency time-lapse imaging of seedlings of *icr2-2×UBQ10::RFP-MBD×ICR2-3×YPet* at 8 DAG. Seedlings were grown on CellView 35/10 mm glass-bottomed cell culture dishes (Greiner, 627860) at a 45° angle, so that roots grew along the glass bottom between the growth medium and the glass. Imaging of root hairs was done by taking a *z*-stack of ten focal planes at 5-min intervals, for a total of 30 frames, using an LSM 780-NLO confocal laser scanning microscope (Zeiss) with a 63× water immersion objective. Images were de-noised using ND-Safir software (Boulanger et al., 2010).

### Light and confocal laser scanning microscopy

Stereomicroscopy imaging was preformed using AxioZoomV16 stereomicroscope (Zeiss) with Objective Plan-NEOFLUAR Z 1.0×/0.25 FWD 56 mm. Bright-field and DIC imaging were performed with an Axioplan-2 imaging microscope (Zeiss) equipped with an Axio-Cam and a cooled charge-coupled device camera using either 10×, 20× dry or 63× water immersion objectives with numerical aperture values of 0.5, 0.9 and 1.2, respectively. Laser scanning confocal microscopy and associated bright-field and DIC imaging were performed using an LSM 780-NLO confocal laser scanning microscope (Zeiss) with 10× and 20× air objectives and 40× and 63× water immersion objectives with numerical apertures of 0.3, 0.8, 1.2 and 1.15, respectively. Fluorescein was visualized by excitation with an argon laser at 488 nm; emission was detected between 493 and 556 nm. Rhodamine was visualized by excitation with an argon laser at 561 nm; emission was detected between 566 and 685 nm. 3×YPet was visualized by excitation with an argon laser set at 514 nm; emission was detected 526 and 570 nm. For unmixing, emission was detected with GaAsP spectral detector. Spectral separation was used with 514 nm laser excitation and emission set between 521-690 nm with 8.9-nm step intervals. VND6-induced cultured cells were observed using an inverted fluorescence microscope (IX83-ZDC, Olympus) fitted with a confocal unit (CSU-W1, Yokogawa), a sCMOS camera (ORCA-Fusion, Hamamatsu Photonics), a UPLANSAPO 60× water immersion objective (NA=1.20, Olympus), and laser lines set at 488 and 561 nm. Images were acquired using MetaMorph (Molecular Devices) and analyzed using ImageJ. For ICR2/TUB6 ratio, the region of interest (ROI) for each microtubule was manually selected and the mean intensity within the ROI was used after subtracting background intensity. A total of 194 microtubules from eight cells were analyzed.

### Image analysis

Image analyses were performed with ZEN 2012 Digital Imaging (Zeiss), Photoshop CS5.1 (Adobe Systems) and ImageJ (Fiji).

### Quantification and statistical analyses

Stacked charts and box plots were prepared using JMP (SAS) or Office Excel 2016 (Microsoft). Statistically significant differences were determined using one-way ANOVA with Tukey’s HSD post hoc analysis, as noted in the figure legends and in Tables S2 and S3.

### Microtubule co-sedimentation assay

Porcine brain tubulin was purified as described (Castoldi and Popov, 2003). For the co-sedimentation assay, 0, 1, 2, 4, 6, 8 or 10 μM of purified ICR2-His_6_ was added to taxol-stabilized microtubules (5 μM tubulin) in PEMT (100 mM PIPES, 1 mM EGTA, 1 mM MgCl_2_, 1 mM GTP and 20 µM taxol, pH 6.9). The samples were centrifuged at 100,000 ***g*** at 25°C for 15 min. Pellets and supernatants were analyzed by 10% SDS-PAGE and visualized by staining the gels with Coomassie Brilliant Blue R 250. His-NtMAP65-1c and BSA were used as positive and negative controls, respectively.

### Microtubule immunofluorescence colocalization and *in vitro* bundling assays

Rhodamine-labeled tubulin was prepared as previously described (Hyman, 1991). For the colocalization assay, taxol-stabilized microtubules composed of tubulin mixed with rhodamine-labeled tubulin (molar ratio 1:4) in PEMT were incubated with 0.5 μM ICR2-His_6_ for 15 min at 37°C and then crosslinked with 20 mM 1-ethyl-3-(3-dimethylaminopropyl) carbodiimide (Pierce Biotechnology) for 5 min at 37°C. The mixture was then centrifuged at 12,000 ***g*** for 5 min, and the pellet was resuspended in PEM buffer preheated to 37°C. ICR2-His_6_ was stained with an anti-His antibody (Sigma-Aldrich, H-1029, 1:5000) and a secondary antibody conjugated with fluorescein (Sigma-Aldrich, F0257, 1:5000). The solution was then centrifuged at 12,000 ***g*** for 5 min, and the pellet was resuspended with PEM buffer preheated to 37°C. An aliquot of 1 μl was put on a poly-L-lysine-coated glass slide (Sigma-Aldrich, P0425) and observed by confocal microscopy. For the *in vitro* bundling assay, the same taxol-stabilized rhodamine-labeled microtubules were incubated with 0.1, 0.5, 1 or 2 μM ICR2-His_6_ for 30 min at 37°C and then treated with 0.005% glutaraldehyde. A 1-μl aliquot of each sample was put on a poly-L-lysine-coated glass slide (Sigma-Aldrich, P0425) and observed by confocal microscopy.

## Supplementary Material

10.1242/develop.200811_sup1 Supplementary information
