# Correction: Microtubule-associated ROP interactors affect microtubule dynamics and modulate cell wall patterning and root hair growth

**DOI:** 10.1242/dev.201502

**Published:** 2022-12-14

**Authors:** Gil Feiguelman, Xiankui Cui, Hasana Sternberg, Eliran Ben Hur, Takeshi Higa, Yoshihisa Oda, Ying Fu, Shaul Yalovsky

There were errors in *Development* (2022) **149**, dev200811 (doi:10.1242/dev.200811).

In the Results section and in the title of Fig. S7, the *icr2 icr3 icr5* triple mutant was mentioned in error. No data are shown for this mutant. The corrected text is shown below and the online full-text, PDF and print versions of the article have been updated.

**Corrected:**

The creation of *icr2* and *icr5* single- and double-mutant plants enabled analysis of the functions of these ICRs in MX pit formation (Fig. 1).

**Original:**

The creation of *icr2* and *icr5* single- and double-mutant plants, as well as the *icr2 icr3 icr5* triple mutant, enabled analysis of the functions of these ICRs in MX pit formation (Fig. 1).

**Corrected:**

As ROP signaling plays a central role in root hair tip growth (Bloch et al., 2005, 2011; Carol et al., 2005; Chai et al., 2016; Denninger et al., 2019; Duan et al., 2010; Jones et al., 2002; Kang et al., 2017; Molendijk et al., 2001; Nakamura et al., 2018;Wan et al., 2017), we asked whether single and double ICR mutants develop abnormal root hairs

**Original:**

As ROP signaling plays a central role in root hair tip growth (Bloch et al., 2005, 2011; Carol et al., 2005; Chai et al., 2016; Denninger et al., 2019; Duan et al., 2010; Jones et al., 2002; Kang et al., 2017; Molendijk et al., 2001; Nakamura et al., 2018;Wan et al., 2017), we asked whether single, double and triple ICR mutants develop abnormal root hairs.

**Corrected:**

**Fig. S7. Root hair initiation sites, density, and length in WT and single and double mutants.**

**Original:**

**Fig. S7. Root hair initiation sites, density, and length in WT and single, double, and triple mutants.**

The authorsapologise for these errors and any inconvenience they may have caused.

