# Supplementary material for: Microtubule-associated ROP interactors affect microtubule dynamics and modulate cell wall patterning and root hair growth
Source: Development. 2022 Nov 16;149(22):dev200811. doi: 10.1242/dev.200811 (PMC9845754; doi:10.1242/dev.200811)
Supplement: Supplementary information [file develop-149-200811-s1.pdf]

Supplementary Materials and Methods

Multiplex genome editing design and constructs. The polycistronic tRNA-gRNA system (PTG) was used to generate multiple sgRNAs with different target sequences by flanking the sgRNAs with a tRNA precursor sequence A pJET-gRNA-tRNA plasmid, which contains a gRNA-tRNA-fused fragment, was used as a template to synthesize the PTG construct. The gRNA scaffold fragment was amplified by PCR using a pair of specific primers (Bsa-gRNA-F and gRNA-R), whereas the $tRNA_{Gly}$ fragment was amplified as an overlapping fragment of the primers g-tRNA-F and tRNA-R. Then these two fragments were fused as a gRNA-tRNA by overlapping extension PCR using primers Bsa-gRNA-F and tRNA-R. The overlapping PCR product was separated and purified from an agarose gel, and then inserted into *pJET1.2* (Thermo Scientific) to generate the template plasmid. The specific spacer sequences targeting *ICR2*, *ICR3*, and *ICR5* were selected using the CRISPR-PLANT database (www.genome.arizona.edu/crispr/). The PTG clones were created using Golden Gate (GG) for the assembly of DNA fragments. To ligate multiple DNA fragments in a desired order, GG assembly requires distinct 4-bp overhangs to ligate two DNA fragments after digestion with BsaI. The gRNA spacers are the only unique sequences in the PTG and were used for this purpose. Each part was amplified with spacer-specific primers containing the BsaI adaptor, except two terminal parts using gRNA spacer primer and terminal specific primers containing BbsI site. These PCR fragments were ligated together using GG assembly to produce the PTG with complete gRNA spacers targeting *ICR2*, *ICR3*, and *ICR5*. The assembled product was amplified with short terminus specific primers containing the BbsI adaptor. Next, using a second GG assembly step, the PTG fragment was inserted into the BbsI digested *pEntr_L1L2_AtU6gRNA*. The PTG cassette was than inserted into *pMR294_pKGCAS9PLUS-1* by Gateway LR Clonase (Thermo Fisher Scientific). The *pEntr_L1L2_AtU6gRNA* and *pMR294_pKGCAS9PLUS-1* vectors were gifts from Professor Gitta Coaker, University of California, Davis.

In all cloning, PCR-generated fragments were sequenced to verify that no PCR-generated errors were introduced. In the cases of gene fusions, following cloning, the borders between fragments were sequenced to verify that fragments were in frame. All primers and plasmids used and generated in this work are listed in Tables S4-S6.

Development • Supplementary information

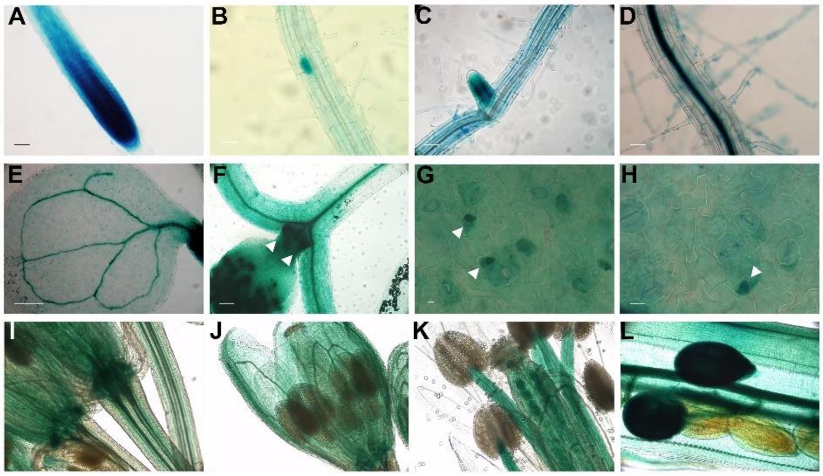

Fig. S1. The expression pattern of ICR2. Expression of ICR2 was analyzed in *pICR2::ICR2-GUS* plants. Expression was detected in (A) the root tip, (B) lateral root initials, (C) developing lateral root, (D) stele and root hairs of root differentiation zone, (E) vascular tissues and developing stomata in cotyledons, (F) vasculature tissues and developing leaves hypocotyl (ICR2 is indicated by arrowheads), (G and H) meristemoids and developing guard cells (ICR2 is indicated by arrowheads), (I) vasculature in pedicels and receptacles, (J) vascular tissues in in sepals, (K) the stamen filaments, and (L) ovules, developing seeds, and siliques. Scale bars correspond to 50 μm for panels A-F and 20 μm for panels G-H.

Development • Supplementary information

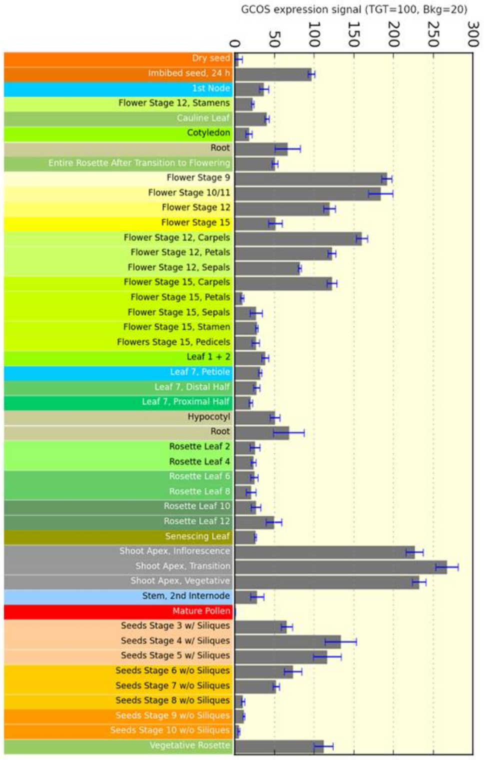

Fig. S2. *ICR2* expression pattern and during development. Transcriptomics data of *ICR2* expression levels. Figure adapted from the *Arabidopsis* eFP Browser (https://bar.utoronto.ca/efp/cgi-bin/efpWeb.cgi).

Development • Supplementary information

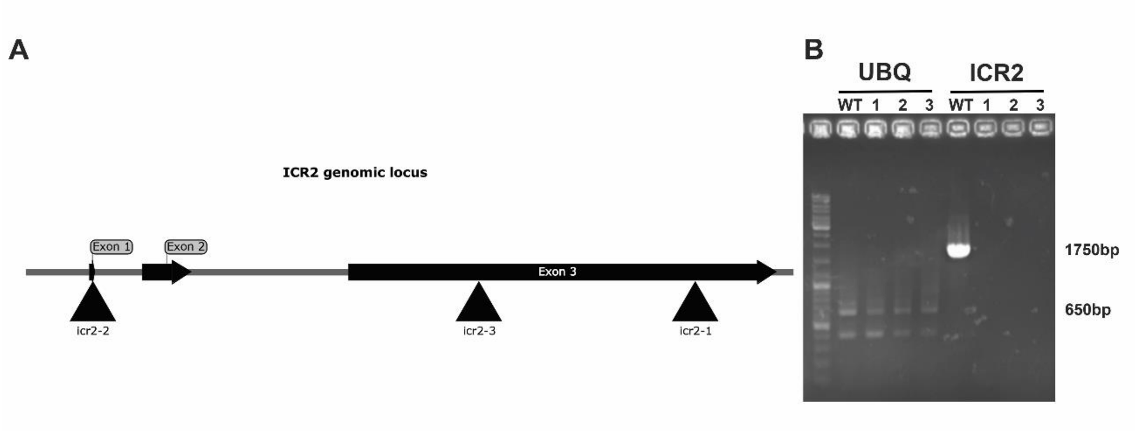

Fig. S3. Map of the *ICR2* locus showing three *icr2 T-DNA* insertion mutant alleles. (A) *icr2-1* (*GK567F02*) *T-DNA* insertion is in the third exon, 297 bp before the stop codon; *icr2-2* (*GK281B01*) insertion is in the first exon, 19 bp after the initiation codon; *icr2-3* (*GK159B08*) insertion is in the third exon, 472 bp after the start of the exon. (B) *icr2* mutant plants have no *ICR2* mRNA transcript (1,750 bp). *Ubiquitin 5* (UBQ) was detected as a control (650 bp).

Development • Supplementary information

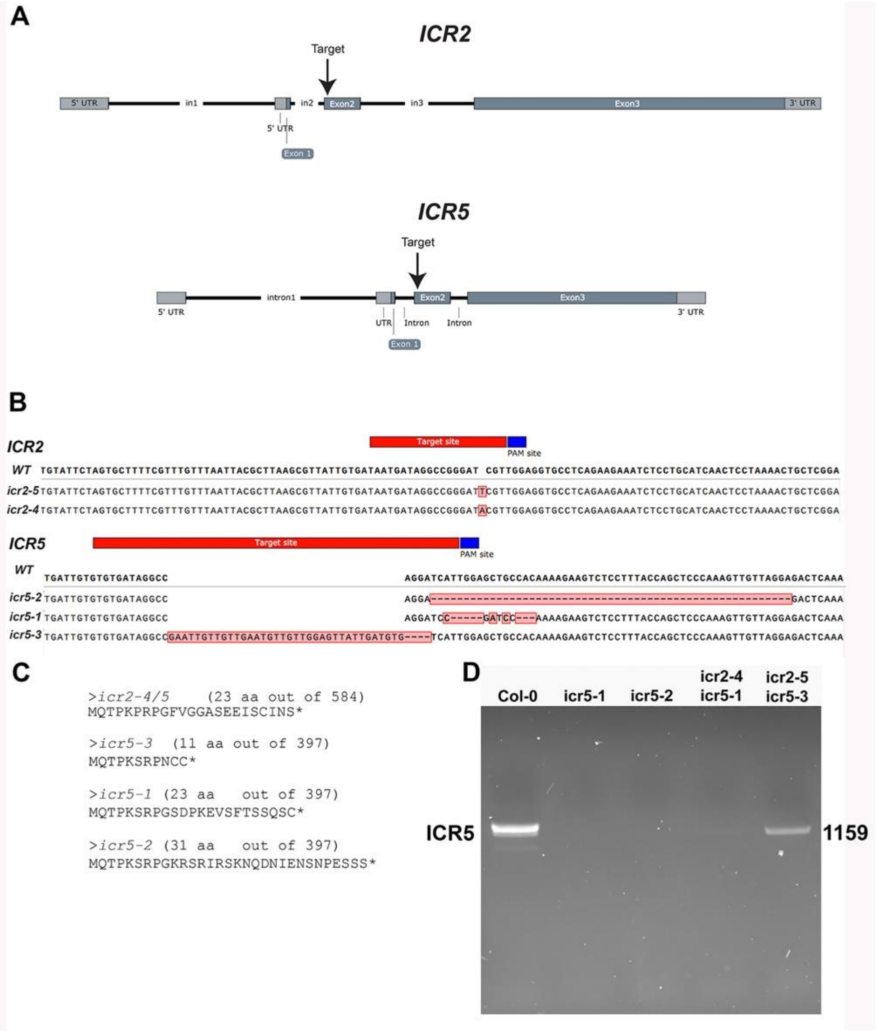

Fig. S4. *The CRISPR/Cas9 generated mutations in ICR2 and ICR5.* (A) Positions of the gRNA target sequence for each gene. (B) Sequences of InDels in the mutant alleles aligned with the WT *Col-0* allele. Inserted bases are marked in red. Dashed lines indicate deletions. (C) Predicted amino acid sequences of the mutants. Asterisk indicates stop codon. (D) RT-PCR of *ICR5* 1,159 bp fragment in Col-0 and the *icr5* CRISPR mutant alleles.

Development • Supplementary information

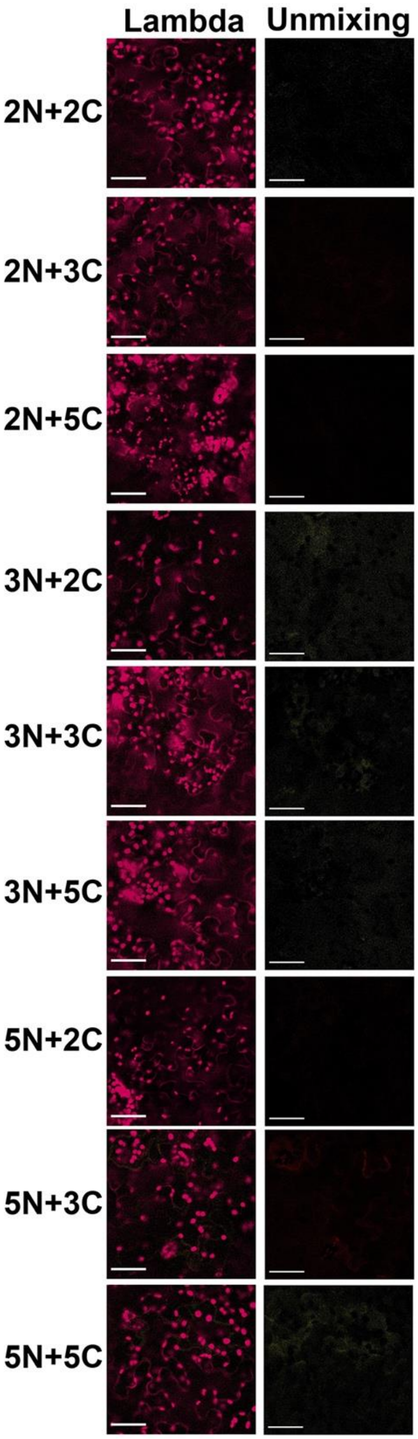

Fig. S5. ICR2 ICR3 and ICR5 do not form homo and hetero dimers when transiently co- expressed in *N. benthamiana* leaf epidermis. ICR2 ICR3 and ICR5 fused to N-terminus of YFP (N) or C-terminus of YFP (C). Images show lambda stacks excited by 514 nm laser and emission collected from 521 nm to 690 nm. The stacks were then unmixed for dye separation of YFP signal. Scale bars, 50 µm. Experiments were repeated twice.

Development • Supplementary information

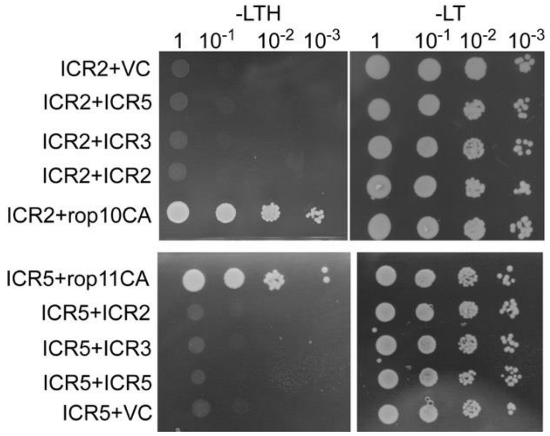

Fig. S6. ICR2 and ICR5 did not homodimerize or heterodimerize with one another and with ICR3 in yeast 2-hybrid assays. Dilution series: 1, 1/10, 1/100, and 1/1,000 yeast 2-hybrid assays. No growth of ICR2 and ICR5 homodimers and heterodimers with each other and with ICR3 was detected on selective -Leu Trp, His (-LTH) media plates supplemented with 1 mM 3-AT. -LT: -Leu, Trp control plates. VC: empty vector control. Interaction assays with constitutively active rop10CA or rop11CA served as a positive controls.

Development • Supplementary information

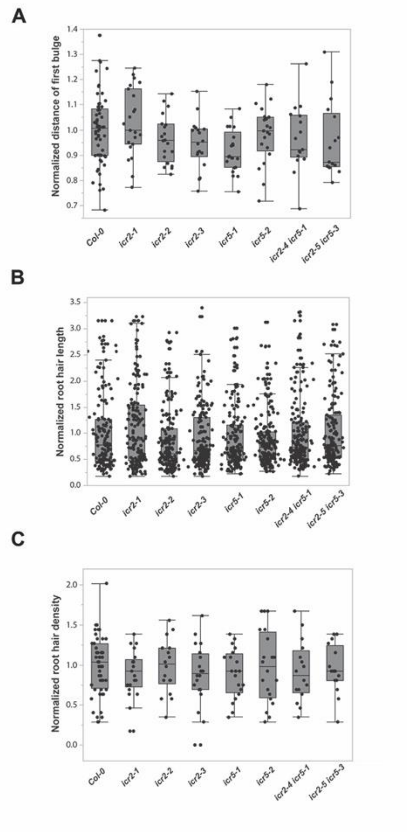

Fig. S7. Root hair initiation sites, density, and length in WT and single and double mutants. Quantification of (A) normalized distance of first bulge from root tip (n≥16 roots), (B) length of root hairs (n≥142 root hairs), and (C) density of root hairs (n≥16 roots). No significant differences were identified between the lines using ANOVA. The boxes are the interquartile ranges, the whiskers represent the 1st and 4th quartiles, and the lines are the averages. See table S16 for Fig. S7A,B and C.

Development • Supplementary information

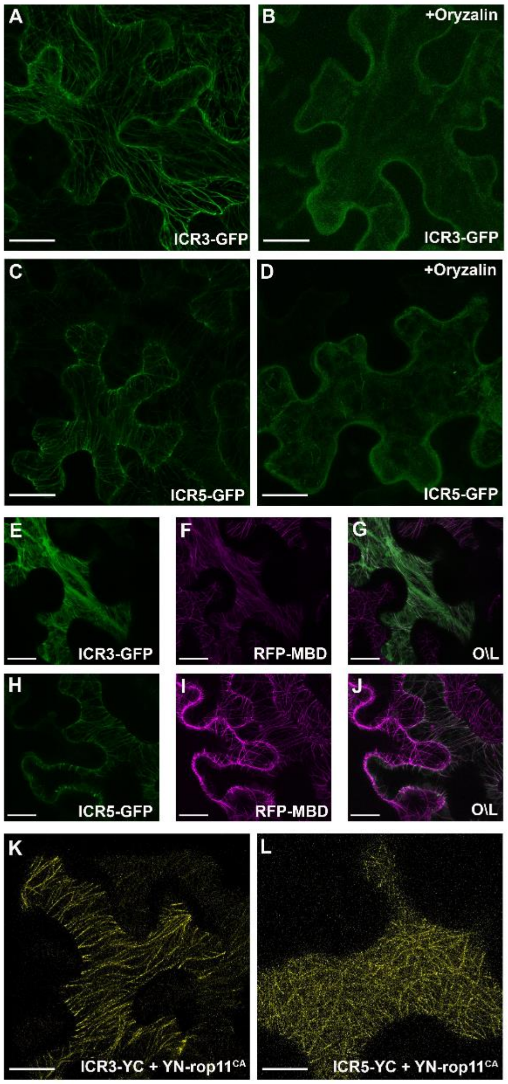

Fig. S8. ICR3 and ICR5 localize on microtubules and interact with ROPs. Distribution of ICR3-GFP and ICR5-GFP transiently expressed in *N. benthamiana* leaf epidermis pavement cells were localized in mock treated (A, C) and following microtubule depolymerization with oryzalin (B, D). Transient co-expression of ICR3-GFP and RFP-MBD (*E* and *F*). Transient co expression of ICR5-GFP and RFP-MBD (H-J). BiFC images of *N. benthamiana* leaf epidermis that transiently express YN-rop11^{CA} with ICR3-YC (K), and YN-rop11^{CA} with ICR5-YC (L). Scale bars are 20 µm.

Development • Supplementary information

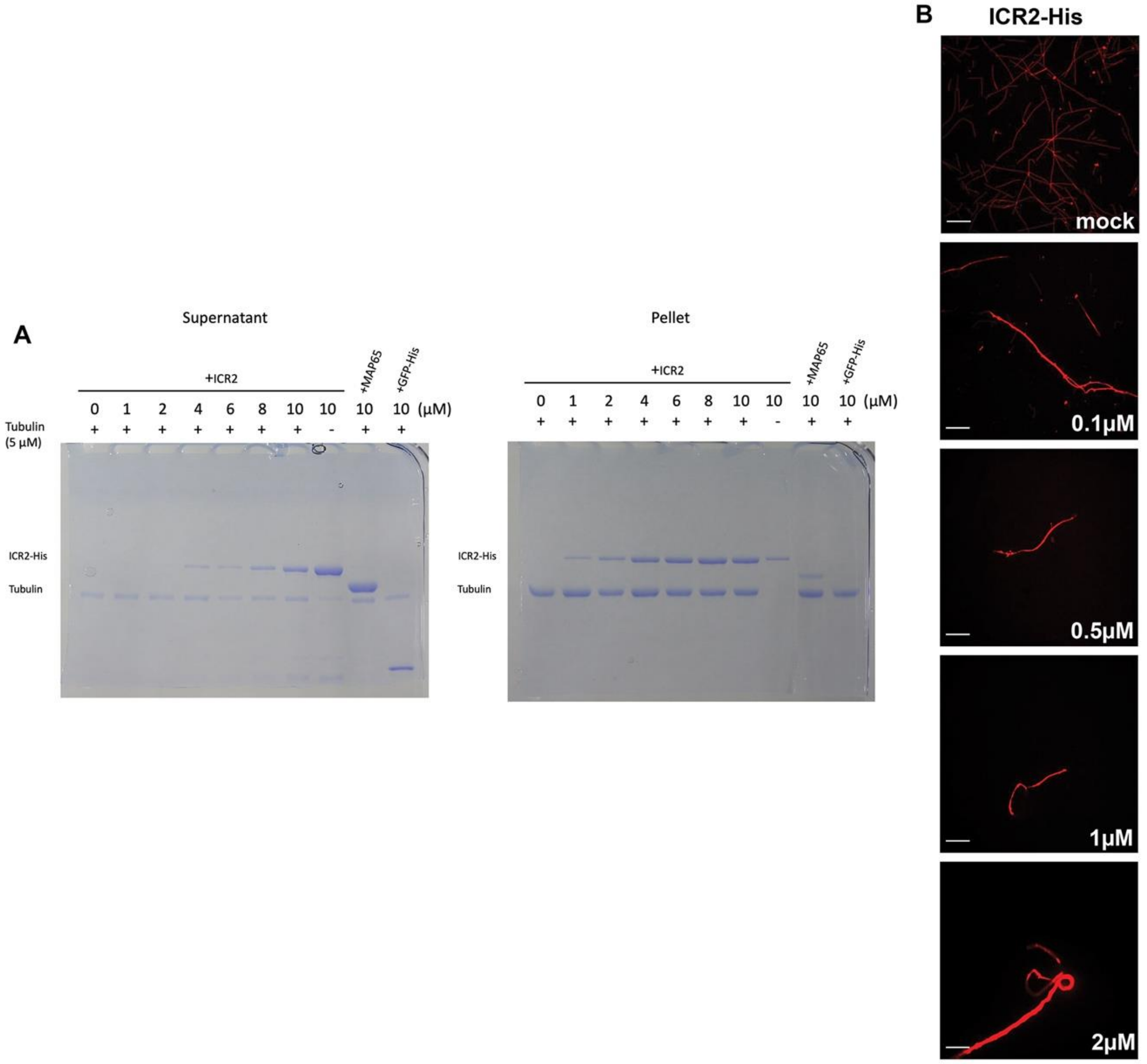

Fig. S9. ICR2 interacts with microtubules in vitro. (A) The full-lengths gels of supernatant (left) and pellet (right) fractions of microtubule-ICR2 co-sedimentation assays shown in Fig. 6*A*. (B) ICR2 induces microtubule bundling. Rhodamine-labeled microtubules (red) formed bundles when incubated with a range of concentrations of recombinant ICR2-His$_6$. The panels displaying mock, 0.1 μM ICR2, and 2 μM ICR2 samples are duplicates of Fig. 6*I*, *J*, and *K*, respectively, and are presented here for the sake of clarity.

Development • Supplementary information

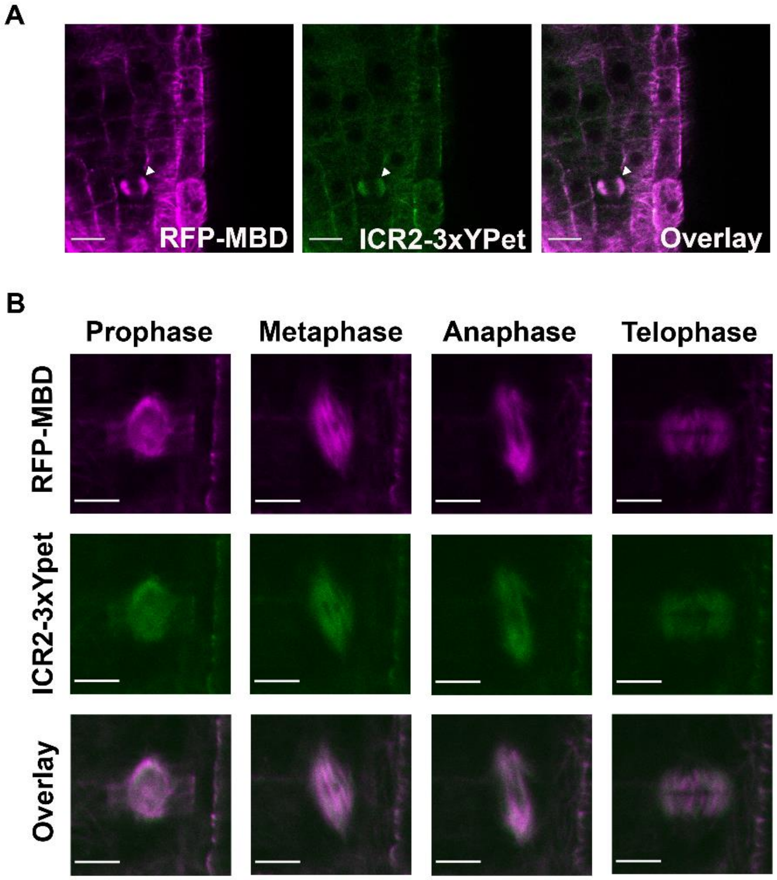

Fig. S10. ICR2-3xYPet co-localizes with microtubules during all stages of cell division. (A) Image of lateral root cap of *icr2-2* plant expressing ICR2-3xYPet (green) and RFP-MBD (magenta). The expanding phragmoplast is indicated by arrowheads. Scale bars, 5 µm. (B) Tracking of a single cell undergoing cell division and cytokinesis in *icr2-2* plant expressing ICR2-3xYPet (green) and RFP-MBD (magenta). Co-localization is observed in all mitotic stages: preprophase band in prophase, spindle during metaphase and anaphase, and phragmoplast in telophase. Scale bars, 5 µm.

Development • Supplementary information

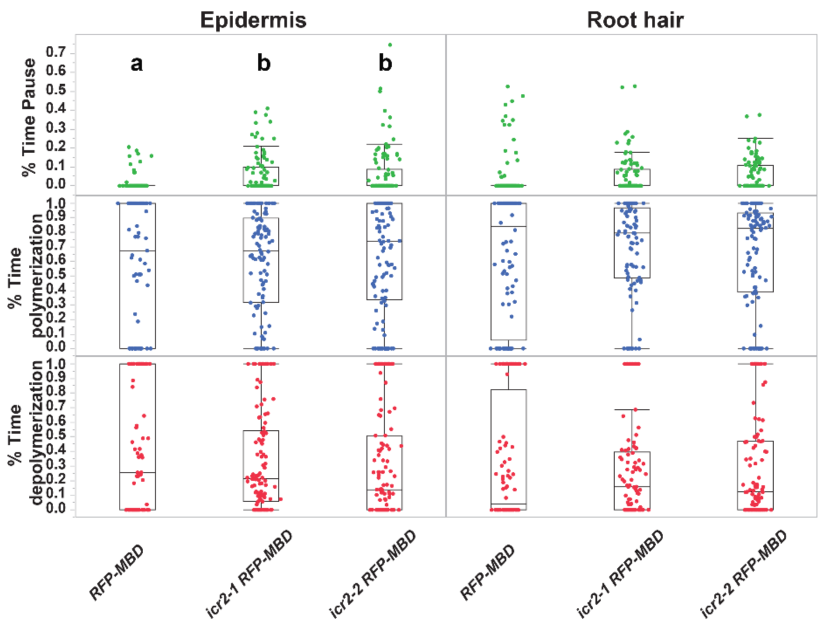

Fig. S11. ICR2 affects pause time of microtubules between growth and shrinkage. Fraction of the time microtubule filaments were extending (blue), pausing (green) or shrinking (red). Means with different letters are significantly different (Tukey's HSD, $p<0.05$). One-way ANOVA results are in Table S3. The boxes are the interquartile ranges, the whiskers represent the 1st and 4th quartiles, and the lines are the averages. n≥77 for each genotype. The imaging was carried out in multiple sessions. See table S15 data for Fig. S9.

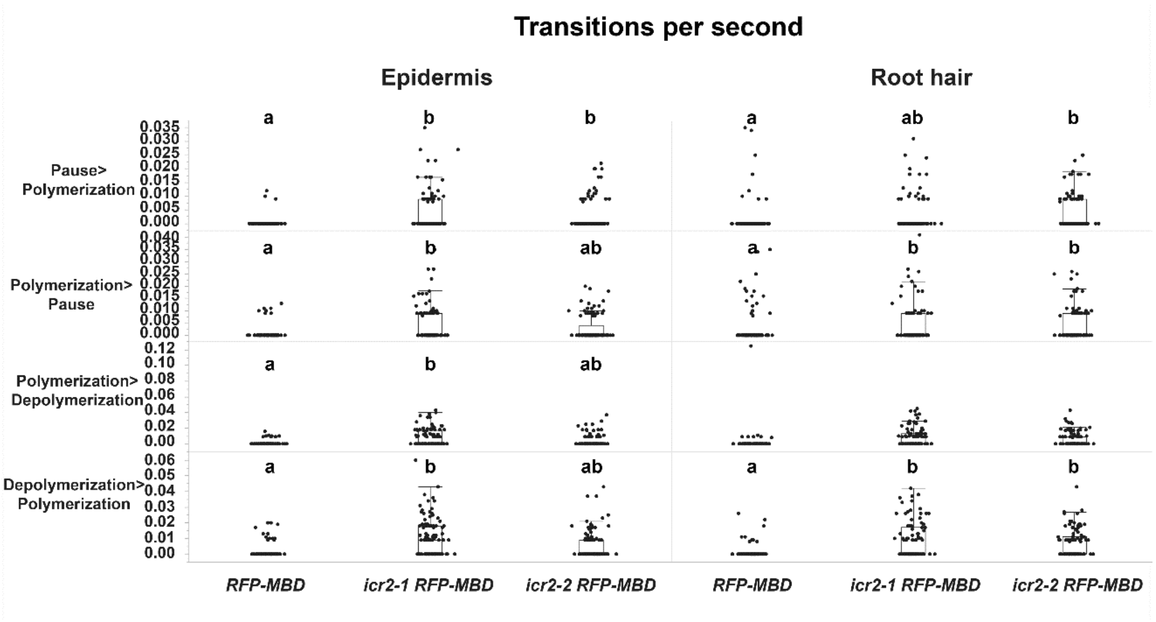

Fig. S12. The effects of ICR2 on microtubule dynamics as detected by frequency of transitions. Number of transitions per second for each genotype at each cell type. In, shrinkage; Out, extension, Pause, pause. Means with different letters are significantly different (Tukey's HSD, $p<0.05$). One-way ANOVA values are in Table S3. The boxes are the interquartile ranges, the whiskers represent the 1st and 4th quartiles, and the lines are the averages. n≥77 for each genotype. Imaging collected in multiple sessions. See table S15 for Fig. S10.

Development • Supplementary information

Table S1. Co expression analysis of ICR2

Click here to download Table S1

Table S2. Description of statistical tests used in this work

Figure	Experiment	Sample size (n)	Sample composition	Repeats	Statistical test	F(df),p value	comments
1M	Pit area	≥101	pits	2*	One way ANOVA + Tukey HSD	F(10, 1716)=38.1919,p<0.0001	*Full experiment (all genotypes) conducted once
1N	Pit density per area	≥10	metaxylem cells	2*	One way ANOVA + Tukey HSD	F(10, 137)=9.2886, p<0.0001	*Full experiment (all genotypes) conducted once
2B	Protoxylem lignification	10	5 roots, 2 px cells per root	2	One way ANOVA + Tukey HSD	F(8, 86)=34.4972, p<.0001	
3B	Root hair morphology	8	8 roots per genotype; 368≤ Root hairs per genotype	2*	N.A.	N.A.	*Oryzlin treatment conducted once
6B	Microtubule co-sedimentation assay		protein bands	3	N.A.	N.A.	Just averaging of the repeats
10,S11, S12	Microtubules dynamics	≥77	MT filaments	N.A.	One way ANOVA + Tukey HSD	See table S3	Collected in multiple sessions

Development • Supplementary information

Table S3. Additional F statistic and p values for ANOVA

Figure	Parameter	F(df),p value	F(df),p value
**10C**		**epidermis**	**root hair**
	Velocity polymerization	F(2, 233)=10.1772,p<0.0001	F(2, 233)=22.8589,p<0.0001
	Velocity depolymerization	F(2, 191)=6.648,p=0.0016	
**S11**		**epidermis**	**root hair**
	Pause	F(2, 289)=5.6446,p=0.0039	
**S12**		**epidermis**	**root hair**
	depolymerization> polymerization _(sec-1)	F(2, 289)=20.1987,p<0.0001	F(2, 282)=19.1902,p<.0001
	polymerization> depolymerization _(sec-1)	F(2, 289)=18.4348,p<0.0001	F(2, 282)=7.9873,p0.0004
	polymerization >Pause_(sec-1)	F(2, 289)=8.6843,p=0.0002	
	Pause> polymerization _(sec-1)	F(2, 289)=10.3267,p<0.0001	F(2, 282)=3.4118,p0.0343

Table S4. Primers designed during this study

Click here to download Table S4

Table S5. Additional primers used during this study

name	Description	sequence
psy1551	ICR2-exon-LP (GABI_567F02)	TCAGTGGAAGAGCTCAAGG
psy1552	RP (GABI_567F02)	CACGATAGGCAACAAAAACATG
psy1553	icr2 3' utr LP	GGATACTCGTCGATGAAAACG
psy1554	icr2 RP	CCACATGGACTAAGTGCTTTG
psy1566	exon ICR2	GATGGTATGAAGATGTCTGAAGCTTG
psy1567	5' ICR2 (cDNA)	CTCTAATCTCAAGCAATGCAGACTCC
-20	general forward primer (gateway)	GTAAAACGACGGCCAG
M13R	general reverse primer (gateway)	TGCCAGGAAACAGCTATGAC
GFP 60 R	GFP reverse primer	cgtcgccgtccagctcgac
GFP R new	GFP reverse primer	ggatccactagtgagctcgtc
GFP F Bam	GFP forward primer	ggaatccatggtgagcaagggcgag
Bsa-gRNA-F	tRNA-gRNA template creation	GGAGACCGAGGTCTCGGTTTTAGAGCTAGAAATA
g-tRNA-F	tRNA-gRNA template creation	GCACCGAGTCGGTGCAACAAAGCACCAGTGGTCTAGT GGTAGAATAGTACCCTG
gRNA-R	tRNA-gRNA template creation	GCACCGACTCGGTGCCAC
tRNA-R	tRNA-gRNA template creation	CTGCCATGCACCAGCCGGGAATCGAACCCGGGTCTGT ACCGTGGCAGGGTACTATTCTAC

Table S6. Plasmids created during this study

Click here to download Table S6

Development • Supplementary information

Table S7. Transgenic plant lines created and characterized during this study

Name	Genotype	Line	Resistance	Segregation
AtSY2600	*icr2-1 GABI-KAT GK_567F02*		Sulfadiazine	homozygous
AtSY2601	*pB7-pICR2::ICR2-GUS*	1	Basta	heterozygous
AtSY2602	*pB7-pICR2::ICR2-GUS*	2	Basta	heterozygous
AtSY2603	*pB7-pICR2::ICR2-GUS*	3	Basta	heterozygous
AtSY2604	*pB7-pICR2::ICR2-GUS*	4	Basta	heterozygous
AtSY2668	*icr2-2 GABI-KAT GK281B01*		Sulfadiazine	homozygous
AtSY2669	*icr2-3 GABI-KAT GK159B08*		Sulfadiazine	homozygous
AtSY2670	*UBN::RFP-MBD*		Basta	homozygous
AtSY2671	*icr2-1 x UBN::RFP-MBD*		Sulfadiazine+Basta	homozygous
AtSY2672	*icr2-2 x UBN::RFP-MBD*		Sulfadiazine+Basta	homozygous
AtSY2673	*icr2-1 x UBN::RFP-MBD x pK7-pICR2::ICR2-3xYpet*	*1*	Sulfadiazine+Basta+Kanamycin	homozygous
AtSY2674	*icr2-1 x UBN::RFP-MBD x pK7-pICR2::ICR2-3xYpet*	*2*	Sulfadiazine+Basta+Kanamycin	homozygous
AtSY2675	*icr2-2 x UBN::RFP-MBD x pK7-pICR2::ICR2-3xYpet*	*1*	Sulfadiazine+Basta+Kanamycin	homozygous
AtSY2676	*icr2-2 x UBN::RFP-MBD x pK7-pICR2::ICR2-3xYpet*	*2*	Sulfadiazine+Basta+Kanamycin	homozygous
AtSY2677	*icr5-1 (PTG 169-3-2)*		N.A.	homozygous
AtSY2678	*icr5-2 (PTG 264-3-3)*		N.A.	homozygous
AtSY2679	*icr2-4/icr5-1 (PTG 169-7-2)*		N.A.	homozygous
AtSY2680	*icr2-5/icr5-3 (PTG 228-16-21)*		N.A.	homozygous

Table S8. Materials used in this work

Click here to download Table S8

Table S9. Pit area and density

Click here to download Table S9

Development • Supplementary information

Table S10. Protoxylem coil distance

Click here to download Table S10

Table S11. Root hair morphology

Click here to download Table S11

Table S12. MT co sedimentation

Click here to download Table S12

Table S13. ICR2 MT localization profiles

Click here to download Table S13

Table S14. ICR2 in VND6 cells

Click here to download Table S14

Table S15. MT dynamics

Click here to download Table S15

Table S16. Root hair initiation, length and density

Click here to download Table S16

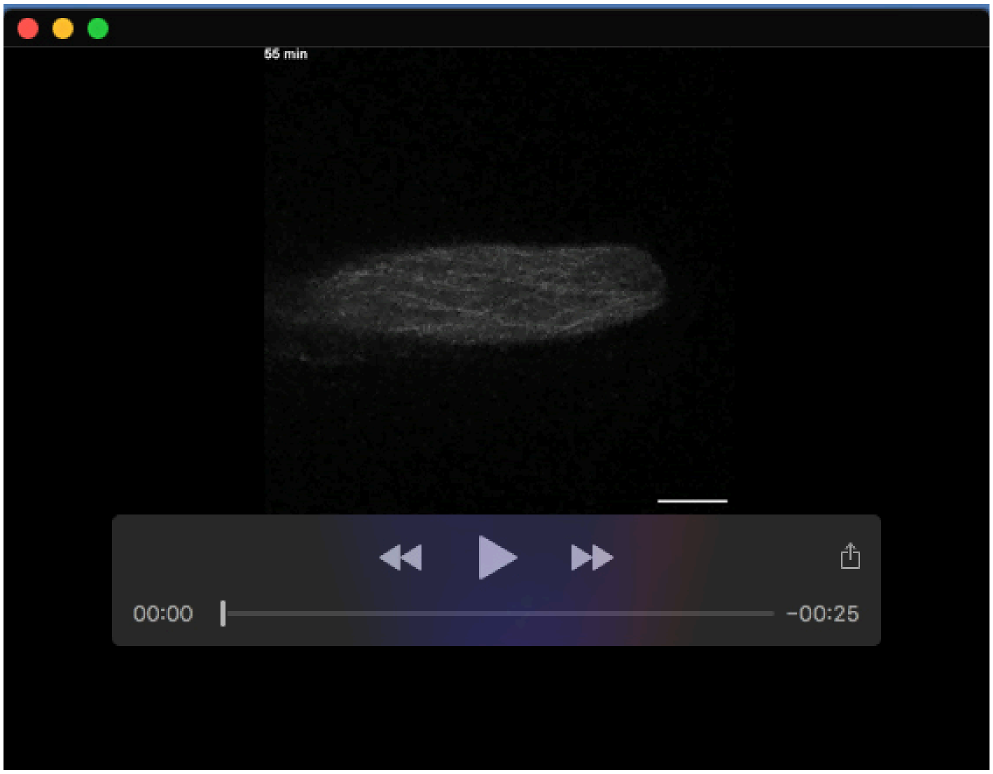

Movie 1. ICR2 in a growing root hair. Video of pICR2::ICR2-3xYPet in a growing root hair. Time listed in minutes. Each image is a maximum intensity projection of 10 focal planes. Images acquired every 5 minutes, Scale bar, 10 µm. Images were de-noised using ND-Safir software (Boulanger et al., 2010).
